# A new piece in the repeatome puzzle of Triatominae bugs: The analysis of *Triatoma rubrofasciata* reveals the role of satellite DNAs in the karyotypic evolution of distinct lineages

**DOI:** 10.1111/imb.13013

**Published:** 2025-06-27

**Authors:** Sebastián Pita, Pablo Mora, José M. Rico‐Porras, Diogo C. Cabral‐de‐Mello, Francisco J. Ruiz‐Ruano, Teresa Palomeque, Ho Viet Hieu, Francisco Panzera, Pedro Lorite

**Affiliations:** ^1^ Evolutive Genetic Section, Faculty of Sciences University of the Republic Montevideo Uruguay; ^2^ Department of General and Applied Biology Institute of Biosciences/IB, UNESP—São Paulo State University Rio Claro Brazil; ^3^ Department of Experimental Biology, Genetics Area University of Jaén Jaén Spain; ^4^ Centre for Molecular Biodiversity Research, Leibniz Institute for the Analysis of Biodiversity Change Bonn Germany; ^5^ Bonn Institute for Organismal Biology – Animal Biodiversity University of Bonn Bonn Germany; ^6^ Department of Medical Microbiology and Parasitology, Faculty of Medicine Duy Tan University Da Nang Vietnam

**Keywords:** fluorescence in situ hybridisation, heterochromatin, kissing bug, repeatome, repetitive DNA, satellite DNA, satellitome, Triatominae

## Abstract

The genome of *Triatoma rubrofasciata*, a representative of the North American Triatomini lineage, was analysed to characterise its repetitive DNA content and satellite DNA (satDNA) organisation. Using RepeatExplorer2, we determined that repetitive elements comprise approximately 25% of the genome in a male sample from Vietnam and 16% in a female sample from China, with satDNA being the most abundant component. The satellitome analysis revealed 126 satDNA families in the male and 114 in the female, with marked quantitative differences driven by the amplification of two satDNA families: TrubSat001‐166 and TrubSat002‐9. Fluorescence in situ hybridization (FISH) confirmed that TrubSat002‐9 is enriched in the Y chromosome, explaining its lesser abundance in the female genome. Chromosomal mapping revealed three distribution patterns of satDNA: (i) localisation in autosomal heterochromatin, (ii) restriction to the Y chromosome, and (iii) presence in euchromatin. SatDNA landscapes showed sharp peaks at low divergence values, consistent with recent amplifications in heterochromatic regions, and broader peaks at higher divergence levels, suggesting older satDNAs located in euchromatic regions. Additionally, several satDNA families are conserved among *T. rubrofasciata*, *T. infestans*, *T. delpontei* and *Rhodnius prolixus*, supporting the “library hypothesis” of satDNA evolution. Our findings highlight the differential amplification of satDNA families linked to heterochromatin expansion, particularly in autosomes, and the conservation of Y‐linked repeats. This study provides new insights into the dynamic role of satDNAs in the karyotypic evolution of Triatominae bugs.

## Graphical abstract

- Satellite DNAs comprise the major component of the *Triatoma rubrofasciata* repeatome, highlighting their central role in genome composition and architecture.
- Satellite DNA families show recent amplification in heterochromatin and older, more divergent satellite DNAs located in euchromatin, indicating distinct evolutionary histories.
- Autosomal satellite DNAs undergo differential expansion across species, while Y‐linked repeats are conserved, reflecting contrasting evolutionary pressures on repetitive DNA.

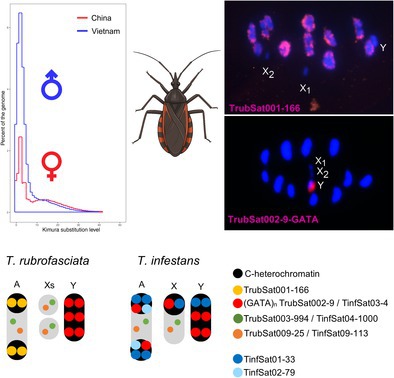

## INTRODUCTION

Triatominae, a subfamily of Reduviidae (Hemiptera, Heteroptera), are traditionally characterised by their blood‐feeding behaviour and the morphological adaptations that enable them to locate hosts and feed on blood (Schofield & Galvão, 2009). Commonly known as kissing bugs, Triatominae are well‐recognised vectors of *Trypanosoma cruzi*, the causative agent of Chagas disease, which is the most serious human parasitic disease in Latin America and affects 6–7 million people worldwide (WHO, 2023). These insects play a fundamental role in the epidemiology of Chagas disease, and their control is key to its prevention and eradication. Triatominae comprise more than 150 species described to date (Gil‐Santana et al., 2022; Zhao et al., 2023). However, a recent proposal redefines the subfamily by incorporating closely related predatory Reduviidae genera, such as *Zeluroides*, *Neivacoris*, *Zelurus* and *Opisthacidius* (Masonick et al., 2024). According to the classic classification, most Triatominae species are found in the Americas. However, two genera are also present in Asia: *Linshcosteus*, with six species, and *Triatoma*, with 10 species (Monteiro et al., 2018; Zhao et al., 2023). Currently, the species of the Triatomine tribe are included in three main lineages: *Triatoma dispar*, North America and South America (Monteiro et al., 2018).

Cytogenetic studies performed in more than 100 species have revealed that Triatominae have a relatively stable karyotype formula, with diploid numbers ranging from 2n = 21 to 2n = 25 chromosomes in males, and three possible sex chromosome systems (XY, X_1_X_2_Y and X_1_X_2_X_3_Y) (Panzera et al., 2021). On the other hand, the constitutive heterochromatin (C‐heterochromatin) content is highly variable among species, revealing intense dynamism for this genomic fraction (Panzera et al., 2021). This variability is evident in the number, size and location of C‐banded regions on both autosomes and sex chromosomes. In autosomes, C‐heterochromatin can be positioned terminally or interstitially, with significant variation in size and abundance among species (Panzera et al., 2021). While the X chromosome is typically euchromatic, certain species exhibit small and infrequent heterochromatic regions. On the other hand, the Y chromosome is entirely heterochromatic across all studied species of Triatominae, thus being a conserved feature in the group. Occurrence of high amounts of heterochromatin has been observed, for example, in *Triatoma nitida* that exhibits C‐heterochromatic blocks in one of its autosomal pairs that can cover up to 80% of the chromosome length. *Triatoma delpontei* is another species with a great amount of C‐heterochromatin, in which all autosomal pairs and the X chromosome, bear C‐heterochromatic blocks that span nearly half their lengths (Panzera et al., 2021). Interestingly, *Triatoma infestans* displays notable intraspecific polymorphism, with populations categorised into Andean and non‐Andean chromosomal groups. Andean populations have approximately 50% more heterochromatin than their non‐Andean counterparts, a difference that correlates with larger genome sizes. On the other hand, several species lack C‐banded regions, except for the Y chromosome. Interestingly, the C‐heterochromatin distribution patterns could be often conserved within closely related species or lineages, reflecting evolutionary trends. This was observed across species within the *T*. *infestans* complex that share similar heterochromatic profiles, with variations as a result of expansion and contraction of C‐heterochromatin blocks linked to speciation and population divergence (Panzera et al., 2014). In this way, it is evident that C‐banding techniques have been fundamental in discovering a wide chromosomal variability in Triatominae, allowing both population differentiation and the identification of new species. For instance, in *Triatoma sordida*, variations in C‐heterochromatin have been key to distinguishing *T. sordida* from *Triatoma rosai* (Panzera et al., 2015). The dynamic nature of C‐heterochromatin, capable of expanding, contracting or relocating within chromosomes, underscores its importance in shaping genome structure and driving evolutionary processes within Triatominae.

*Triatoma rubrofasciata* is the type species for the genus *Triatoma* (Lent & Wygodzinsky, 1979) and was the first species to be described, based on a specimen collected in Indonesia (then known as the Dutch East Indies). Its biogeographical origin, however, has been a topic of long‐standing debate. Despite its phenotypic variability in body colour, which ranges from orange‐marked specimens to almost melanistic forms, several morphometric and genetic studies agree that global populations represent a single species (Dujardin et al., 2015; Gorla et al., 1997; Hieu et al., 2019; Patterson et al., 2001). Furthermore, it is now well established that *T. rubrofasciata* belongs to the so‐called ‘North American’ lineage (sensu Monteiro et al., 2018), along with all other Old World triatomine species. This implies a common ancestry, with the lineage clearly originating in the Americas (Hypša et al., 2002; Kieran et al., 2021; Monteiro et al., 2018; Patterson & Gaunt, 2010). Interestingly, this species has an atypical karyotype for Triatominae, being the only representative with 11 autosomal pairs (reviewed by Panzera et al., 2021). This distinct karyotype is explained by a fusion involving two autosomes, homologous to chromosome 6 of *Rhodnius prolixus* (Mathers et al., 2021). Along with the X_1_X_2_Y sex chromosome system, this results in a male karyotype formula of 2n = 25. In terms of C‐heterochromatin, the Y chromosome is entirely C‐heterochromatic, while the X_1_ and X_2_ chromosomes lack C‐heterochromatin. Autosomes, on the other hand, exhibit conspicuous C‐heterochromatic blocks at both chromosomal ends (Hieu et al., 2019).

Recently, the genome of *T. rubrofasciata* was sequenced, assembled and annotated, making it the second Triatominae genome to be released. Notably, it was the first in the subfamily to achieve chromosome‐level assembly (Liu et al., 2019). However, the estimated genome size (757 Mb) was inaccurate. The authors assumed a genome size similar to that of *R. prolixus* (733 Mb), which was the only Triatominae genome assembled at that time (Mesquita et al., 2015). However, it is widely known that *Rhodnius* species have significantly smaller genomes than those of the genus *Triatoma* (Panzera et al., 2007, 2021). Flow cytometry analyses of 15 *Triatoma* species reveals that they have at least 25% more DNA content compared with the species from the *Rhodnius* genus that were examined (Panzera et al., 2007). Using fluorescence flow cytometry, we have determined that the genome size of *T. rubrofasciata* is about 1187 Mb (unpublished data). Since the BUSCO gene set was nearly complete in the *T. rubrofasciata* assembly, the unplaced DNA sequences are likely composed of repetitive elements. It is well documented that the repetitive nature of these sequences challenge genome assembly, as they can introduce gaps during the process (Ahmad et al., 2020; Tørresen et al., 2019). This issue is exemplified by the model species *Tribolium castaneum*, in which the two major satellite DNA (satDNA) families (TCAST1 and TCAST2) account for approximately 35% of the genome, but when evaluating the genome assembly, the fraction of these repetitive sequences drops to only 0.3% (Pavlek et al., 2015; Wang et al., 2008). A similar case is reported in the satellitome analysis of *R. prolixus*. Using a method that does not depend on a genome assembly, such as RepeatExplorer (Novák et al., 2020), repetitive sequences are estimated to represent about 20% of the genome (Montiel et al., 2021), but only 5.6% when quantified within the assembled genome (Mesquita et al., 2015).

Although the non‐repetitive sequences were well annotated in the genome of *T. rubrofasciata*, the assembly lacks a detailed characterisation of its repetitive fraction (Liu et al., 2019). The collection of repetitive sequences in a genome, known as the repeatome, is mainly composed of transposable elements (TEs), which have the ability to relocate within the genome, and satellite DNAs (satDNAs), which consist of tandemly repeated units (Charlesworth et al., 1994; Mestrović et al., 2015; Palomeque & Lorite, 2008; Pritham, 2009). These sequences were identified and annotated in the genome of *T. rubrofasciata* using the widely adopted tools RepeatModeler and RepeatMasker (Liu et al., 2019). Although these tools are highly effective, careful manual curation is essential to ensure accurate annotation of repetitive elements (Carrasco‐Valenzuela et al., 2025). Especially for satDNAs, the available information is limited, apart from the use of the Tandem Repeat Finder (TRF) software (Benson, 1999). Using these strategies, 55.49% of the genome was classified as repetitive elements (Liu et al., 2019). Additionally, since the assembly was based on a female genome, it precludes the study of repetitive DNA sequences present in the Y chromosome.

Considering the impact of repetitive DNAs on the structure, evolution and functioning of insect genomes (review by Cabral‐de‐Mello & Palacios‐Gimenez, 2025), the characterisation of this fraction is relevant, including non‐model species and those of economic or health interest, such as Triatominae bugs. Given the limited information on repetitive DNAs obtained from the *T. rubrofasciata*'s genome assembly, in this study we aimed to provide a more detailed characterisation of the repetitive DNA content, with a special focus on satDNAs. Moreover, we aimed to deepen the understanding of satellite organisation and evolution between Triatomini lineages as *T. rubrofasciata* belongs to the North American lineage, studied here for the first time. As the fourth Triatomine species analysed in this way (after *T. infestans*, *T. delpontei* and *R. prolixus*), our study provides an additional piece of the complex puzzle of repetitive DNA and heterochromatin organisation in this group. It is worth mentioning that data analysed hitherto have demonstrated that satDNAs serve as the principal architects of heterochromatin, and therefore likely drive the karyotypic evolution observed among Triatomine species (Montiel et al., 2021; Mora et al., 2023; Pita et al., 2018; Pita, Panzera, Mora, et al., 2017). To achieve this, we took advantage of the available Illumina data from the genome assembly project (PRJNA516044, SRR8466737) and generated new genomic data from an individual of different geographic origin and sex. The satDNAs were characterised using a cytogenomic approach, integrating bioinformatic and cytogenetic tools. Our results reveal discrepancies with those obtained in the analysis of repeats in the assembled genome of *T. rubrofasciata*, highlighting the importance of de novo characterisation of repeats using unassembled reads. Moreover, we noticed that satDNA sequences are the main component of the repetitive DNA in Triatomini, including the two main lineages, North and South American. Furthermore, the heterochromatic regions are predominantly composed of satDNA, highlighting the critical role of these sequences in shaping the genome architecture of this species.

## MATERIALS AND METHODS

### Samples, genomic DNA (gDNA) extraction, and repeatome characterisation

Samples of *Triatoma rubrofasciata* were collected from Da Nang, Vietnam. A single male was selected for genomic DNA (gDNA) isolation to ensure the representation of all chromosomes, including the heterogametic X and Y sex chromosomes. Genomic DNA was extracted from the legs and head using the Gentra Puregene kit (Qiagen, Hilden, Germany), following the manufacturer's instructions. Extracted gDNA was sequenced on the Illumina® HiSeq™ 2000 platform by the Macrogen company. The low‐coverage sequencing yielded around 2.5 Gb of 101 bp paired‐end reads. This Illumina library was deposited in the SRA database (accession number SAMN48410004, BioProject PRJNA391552). Low‐quality reads and adapter sequences were removed using *fastp* v0.23.2 using options ‘‐l 150 ‐r ‐n 0’ (Chen, 2023). Cleaned FASTQ files were converted to FASTA format using *seqtk* v1.4 (https://github.com/lh3/seqtk, accessed on 16 July 2024). A random subset of 12 million (6 million of each pair) reads was selected to generate a single FASTA dataset for further analysis. The repeatome was analysed using the RepeatExplorer2 pipeline, which includes TAREAN. Default settings were used, except for computation time (set to extra‐long) and the coverage threshold, which focused on clusters with abundances exceeding 0.001% (Novák et al., 2020). From the input file of 12 million reads, RepeatExplorer2 used a total of 2,710,477 reads (approximately 271 Mb) in the analysis. According to our flow cytometry data, the genome size of *T. rubrofasciata* is approximately 1187 Mb. Therefore, the 271 Mb used by RepeatExplorer2 corresponds to a coverage of about 0.22×, which falls within the recommended coverage range for this type of analysis (0.1 × −0.5×) (Novák et al., 2020). The same approach was applied to Illumina reads from the genome sequencing project (accession number SRR8466737, BioProject PRJNA516044), which corresponds to a female specimen collected in Foshan, Guangdong Province, China (Liu et al., 2019). To facilitate interspecies comparisons, a custom database of known satDNA sequences from *T. infestans*, *T. delpontei* and *R. prolixus* was used (Montiel et al., 2021; Mora et al., 2023; Pita, Panzera, Mora, et al., 2017).

Clusters generated by RepeatExplorer2 and TAREAN, particularly those exhibiting sphere or ring‐like shapes, were further analysed to comprehensively characterise the satDNA families. For satDNAs identified either through manual inspections or by TAREAN, Geneious v.4.8.5 software (Kearse et al., 2012) was used to generate and validate consensus sequences, as well as to determine the monomer size of each satDNA family. Repetitive DNA quantification and divergence values were calculated using RepeatMasker v.4.1.4 (Smit et al., 2013‐2015) by mapping 2 million randomly selected reads back to the consensus sequences. For satDNAs with repeat unit lengths (RUL) exceeding 100 bp, consensus sequences were concatenated into dimers; for RULs shorter than 100 bp, sequences were concatenated into approximately 200 bp fragments. Kimura 2‐parameter (K2P) divergence values were calculated using the “*calcDivergenceFromAlign.pl*” script from the RepeatMasker suite. SatDNA landscapes were visualised using the *ggplot2* R package (Wickham, 2016). SatDNA families were named following a nomenclature similar to that proposed by Ruiz‐Ruano et al. (2016), and ranked in descending order of abundance. A BlastN (http://www.ncbi.nlm.nih.gov/) search was conducted against GenBank with an e‐value threshold of 0.001.

Transposable elements annotation was performed through sequence similarity searches of assembled contigs against GenBank using BlastN and BlastX (http://www.ncbi.nlm.nih.gov/), Repbase via CENSOR (http://www.girinst.org/), and a custom database of transposable elements derived from previous RepeatExplorer2 analyses on several Triatominae species (Montiel et al., 2021; Mora et al., 2023; Pita, Panzera, Mora, et al., 2017). The best hit was taken as the valid classification. In case that none of the methodologies were successful, the cluster remained as unclassified.

### Chromosome preparation and physical mapping by fluorescence in situ hybridisation (FISH)

Chromosome preparations were obtained from adult males collected in Da Nang, Vietnam, the same sampling as the male individual used for DNA extraction for Illumina sequencing and probe manufacturing. Testes were carefully extracted and immersed in distilled water for 45 min to induce osmotic shock. The tissues were then preserved in modified Carnoy's solution (3:1 absolute ethanol: glacial acetic acid) and stored at −20°C until use. Chromosome slides were obtained using the squashing method described in Pita, Panzera, Mora, et al. (2017). Physical mapping of selected satDNA families was performed using FISH, corresponding to the main families (most abundant), along with other minor families (low abundance) selected randomly. Specific oligonucleotides with a length range from 20 to 25 bp were designed for each consensus satDNA sequence using Primer‐BLAST (https://www.ncbi.nlm.nih.gov/tools/primer-blast/) (Table S1). These oligonucleotides were labelled with biotin‐16‐dUTP (Roche) using the Terminal Transferase kit (Roche). The FISH procedure was performed according to Cabral‐de‐Mello and Marec (2021) with minor modifications (Rico‐Porras et al., 2024). Biotin‐labelled probes were detected using Alexa Fluor 488‐conjugated streptavidin (Invitrogen, San Diego, CA) at a concentration of 10 μg/mL. For repeats with a low genome proportion, fluorescent immunological detection was performed using the avidin‐FITC/anti‐avidin‐biotin system with two amplification cycles, as in Rico‐Porras et al. (2024). Hybridised slides were mounted in VECTASHIELD with DAPI (4′,6‐diamidino‐2‐phenylindole) (Vector, Burlingame, CA, USA) for chromosome counterstaining. Images were captured using a BX51 Olympus® fluorescence microscope (Olympus, Hamburg, Germany) equipped with a CCD camera (Olympus® DP70) and processed with Adobe® Photoshop® CS4 v11.0 (Adobe Systems, San Jose, CA, USA).

## RESULTS

**FIGURE 1:**
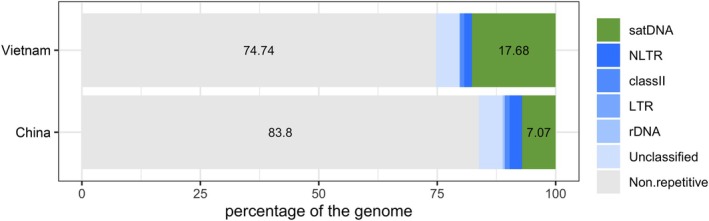
Bar plot representing the genome composition of each *Triatoma rubrofasciata* sample analysed. The X‐axis shows the percentage of the genome. The non‐repetitive fraction is represented in grey. Different shades of blue represent mobile DNA sequences, ribosomal DNA and uncharacterised sequences, while green indicates the fraction of satellite DNA present in the genome. Results are based on RepeatExplorer2 analysis.

The clustering through RepeatExplorer2 revealed that the repetitive content of the *T. rubrofasciata* genome corresponds to about 25% of the DNA content in the male sample from Vietnam, while the repetitive content in the female sample from China is about 16%. Most of these repeats in both genomes were annotated as satDNAs, accounting to 17.68% of the genome composition in the Vietnam sample and about 7.1% in the China sample (Figure 1, Table 1). In contrast with the high abundance of satDNAs, TEs were detected in small proportions, with non‐LTR elements (NLTR) representing 1.62% in the Vietnam sample and 2.63% in the China sample, whereas DNA transposons account for 0.87% and 1.02% in the Vietnam and China genomes, respectively. Long terminal repeat (LTR) elements were identified only in trace amounts (0.02% in both samples) (Figure 1, Table 1). Unclassified sequences accounted for 5% in both samples.

**TABLE 1:** Characterisation of the *Triatoma rubrofasciata* genome using RepeatExplorer2. Values are expressed as genome proportion (%).

Sample	Non‐repetitive	LTR	Non‐LTR	Class II	satDNA	rDNA	Unclassified
Vietnam (male)	74.74	0.02	1.62	0.87	17.68	0.14	4.92
China (female)	83.8	0.02	2.63	1.02	7.07	0.47	4.98

**FIGURE 2:**
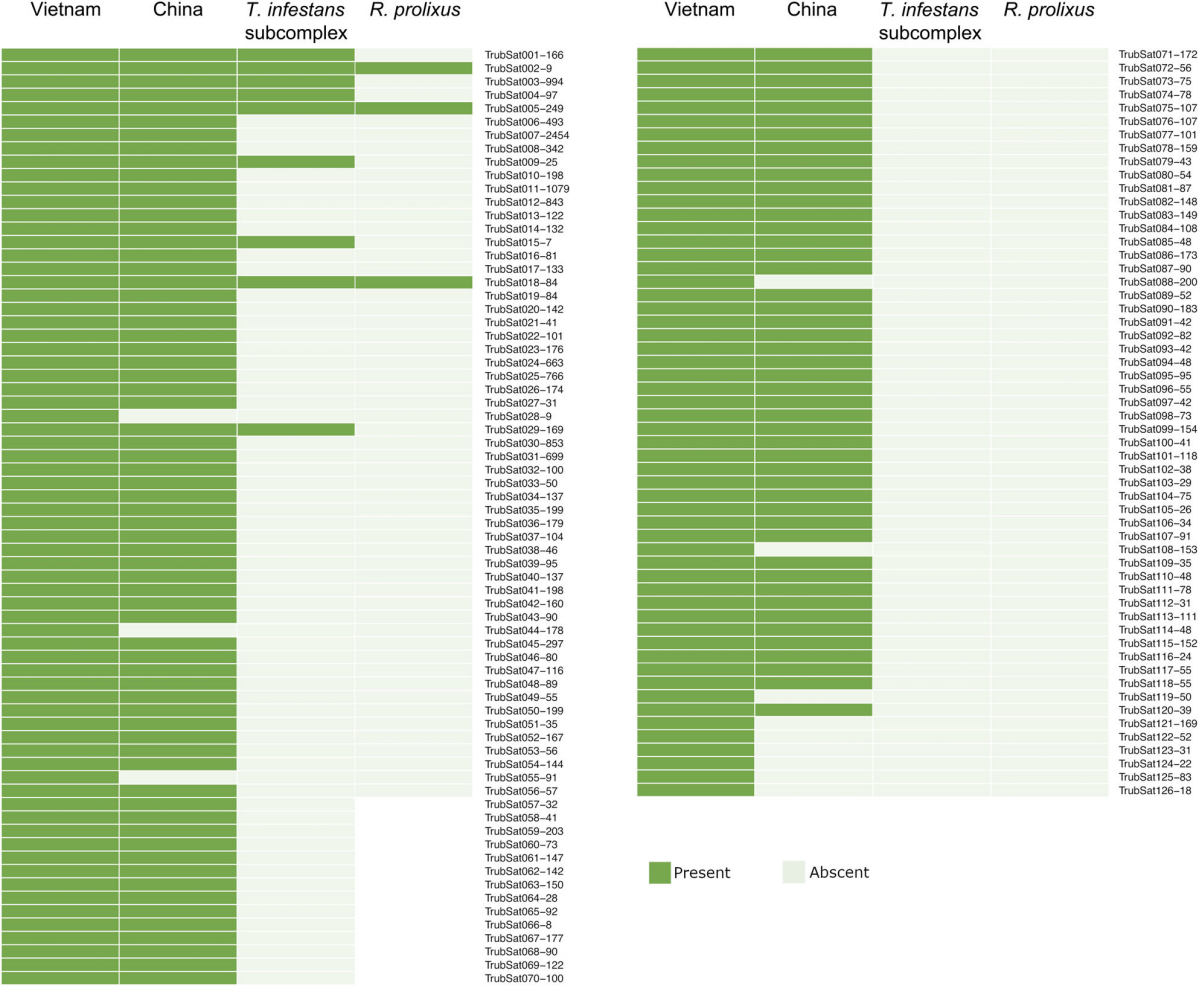
Satellite DNA families shared among *Triatoma rubrofasciata* samples and species from the *Triatoma infestans* subcomplex and *Rhodnius prolixus*.

RepeatExplorer2/TAREAN analyses revealed that the satellitome of the male from Vietnam includes at least 126 different satDNA families, but only 114 families in the female from China (Figure 2, Table S1). This difference is due to the fact that 12 satDNA families detected in the Vietnam sample were not present in the China sample (Figure 2, Table S2); most of them appeared at low frequencies in the male genome. Employing RepeatMasker, all satDNA families from the Vietnam male and China female represented 31.07% and 14.05% of their genomes, respectively. The main differences explaining the variations between both satellitomes are primarily due to the two most abundant satDNA families, TrubSat001‐166 and TrubSat002‐9. TrubSat001‐166 is the most abundant satDNA in both samples; however, in Vietnam, it represents 20.43% of the genome, while in China, it accounts for only 5.48%. On the other hand, TrubSat002‐9 (GATA repeats and its variants, GATAGATTA, GATGGATTA or CATAGATTA) constitutes 3.77% of the genome in the male from Vietnam, but only 0.02% in the female from China (Table S2).

**FIGURE 3:**
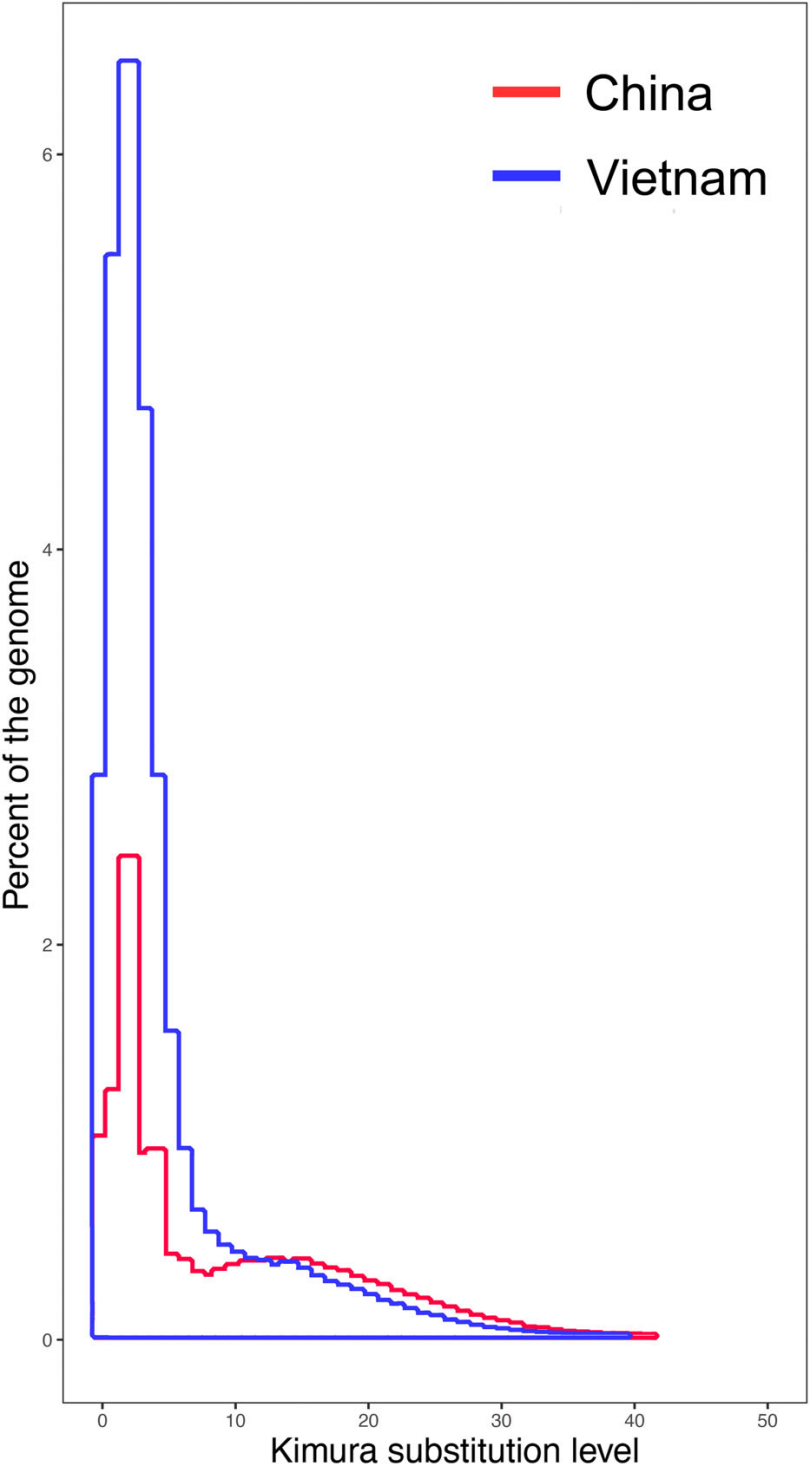
Complete satellite DNA library landscape in the analysed samples of *Triatoma rubrofasciata*. The Y‐axis shows the abundance of repeats as a percentage of the genome, and the X‐axis represents genetic distance corrected using the Kimura 2‐parameter substitution model.

Most of the satDNA families are AT‐rich, with only 9 families having an A + T content below 50%. Analysis of K2P divergence values of satDNA families revealed a range from 0.39% to 28.24% in the Vietnam sample (mean 7.06%). For the China sample, divergence values ranged from 0.45% to 31.97% (mean 8.45%). The relationship between K2P divergence and abundance for satDNAs in each sample, which gives insights into patterns of satDNA evolution (amplification and sequence homogenisation), is shown in Figure 3. Satellitome landscapes revealed a similar pattern in the two samples, although the China sample showed a lower proportion of satDNA, both with a peak of abundance at low K2P divergence values, mainly between 0% and 4%. This peak corresponds mainly to the TrubSat001‐166 satDNA family (Figure S1). The landscapes for each satDNA family in both samples are shown in Figures S2 and S3.

BlastN searches allowed to determine that nine satDNA families of *T. rubrofasciata* had previously been described in other triatomine species of the *T. infestans* subcomplex (*T. infestans* and *T. delpontei*). Three of these families are also present in the *R. prolixus* genome (Figure 2, Figure S4). The similarity between the consensus sequences exceeds 78% in all cases.

**FIGURE 4:**
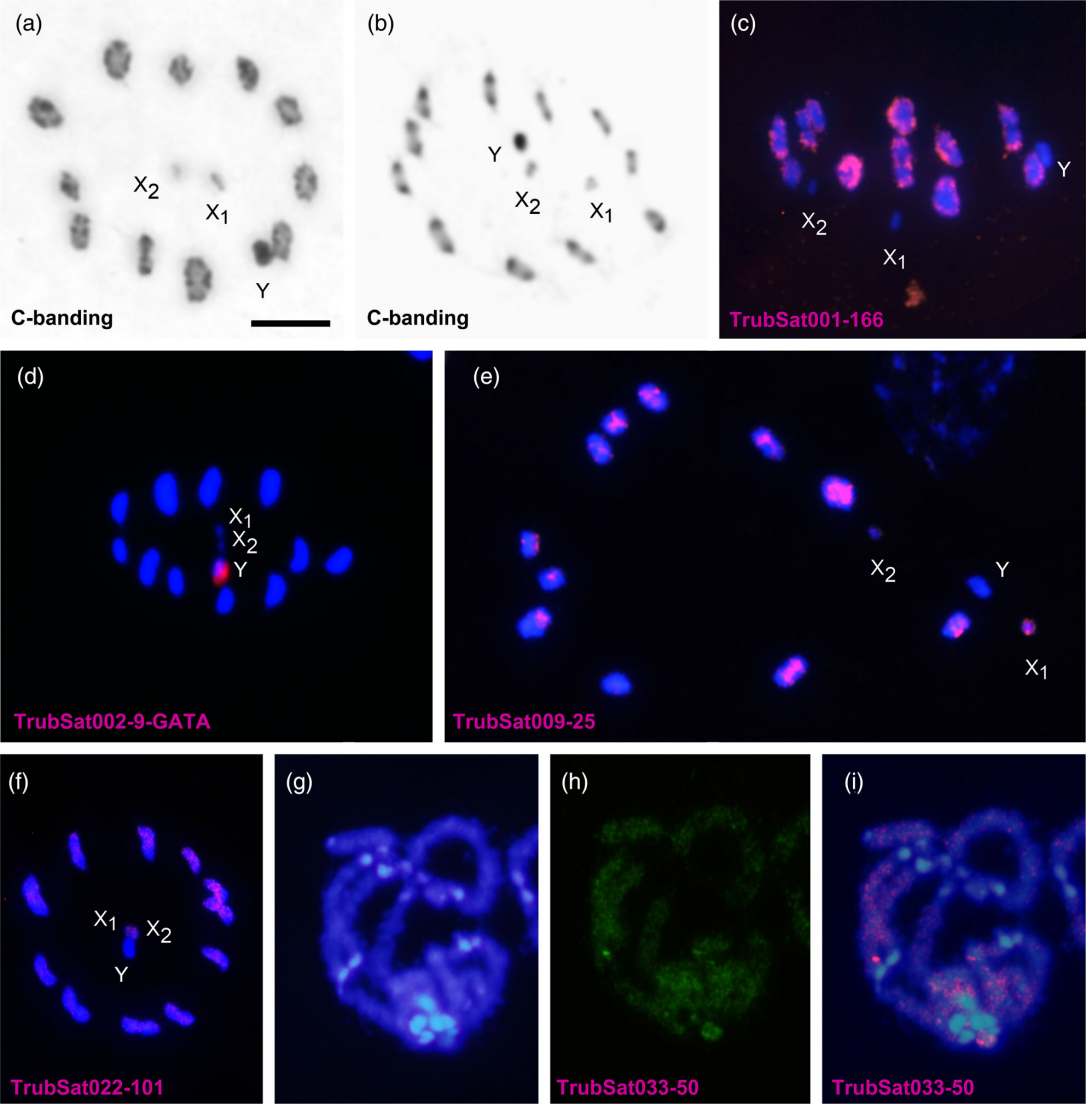
Chromosomal analysis of *Triatoma rubrofasciata*, revealing heterochromatin distribution and location of several satDNAs. C‐banding in meiotic metaphase I (a) and in meiotic metaphase II (b) showing the presence of heterochromatic blocks on the chromosome ends and on Y chromosome. Meiotic metaphase I after hybridisation using TrubSat001‐166 (c), TrubSat002‐9 (d), and TrubSat009‐25 (e) as probes. (f) Meiotic metaphase II after hybridisation using TrubSat022‐101 as a probe. Pachytene stained with DAPI (g), hybridised with TrubSat033‐50 (h), and merged (i).

As the satDNAs were the main component of repetitive DNA sequences in the *T. rubrofasciata* genome, we localised some of them on the chromosomes through FISH, aiming to investigate their enrichment in specific genomic compartments or chromosomes, such as euchromatin/heterochromatin and autosomes/sex chromosomes. Initially, the C‐banding technique was performed to confirm the expected pattern (Hieu et al., 2019). As predicted, the Y chromosome was found to be entirely heterochromatic, and the 11 pairs of autosomes displayed conspicuous bands at both ends (Figure 4a,b). A total of 11 satDNA families were chromosomally located (Table S1). Chromosomal mapping of satDNAs revealed three distinct patterns of distribution: (i) satDNA located in the C‐heterochromatin regions of autosomes (Figure 4c), (ii) satDNA restricted to the C‐heterochromatin of the Y chromosome (Figure 4d), and (iii) satDNA present in euchromatic regions of autosomes and the X chromosome (Figure 4e–i).

## DISCUSSION

In the Triatomini tribe, genomic analyses of repetitive elements, particularly satDNAs, have been limited to representatives of the South America evolutionary lineage (Mora et al., 2023; Pita, Panzera, Mora, et al., 2017). In this study, the analysis of *T. rubrofasciata*, a representative of the North America lineage is carried out, allowing comparisons between both lineages. This approach aims to provide a broader understanding of the organisation and evolution of these repetitive sequences in this insect group.

Here, we advanced in two main aspects: (i) the understanding of the first Triatominae genome assembled at chromosome level, and (ii) the general evolution of satDNAs among Triatomini lineages. The genome assembly of *T. rubrofasciata* reported by Liu et al. (2019) marks a significant milestone as the first chromosome‐level assembly for a Triatominae species. However, our findings reveal discrepancies between our repetitive DNA estimations and those derived from the genome assembly. These inconsistencies may stem from differences in annotation strategies. Specifically, while RepeatModeler and RepeatMasker are effective tools for repeat annotation, their use without posterior curation can lead to misestimation (Carrasco‐Valenzuela et al., 2025; Goubert et al., 2022). Further refinement of the RepeatModeler database, as outlined by Goubert et al. (2022), would be beneficial. Additionally, the analysis by Liu et al. (2019) does not explicitly address satDNA sequences, which have shown to constitute the most significant portion of the *T. rubrofasciata* genome. This highlights the value of non‐assembly‐based approaches, such as RepeatExplorer2 (Novák et al., 2013), TAREAN (Novák et al., 2017), and dnaPipeTE (Goubert et al., 2015), as robust alternatives for characterising the repeatome. These methods analyse short reads directly, bypassing limitations associated with genome assembly quality and enabling for a detailed resolution of repetitive elements. In our study, the combination of RepeatExplorer2 and cytogenetic validation via FISH allowed us to achieve greater accuracy in determining the composition and genomic distribution of satDNA in *T. rubrofasciata*.

The satellitome analysis in one male and one female of *T. rubrofasciata* revealed that most satDNA families are shared across the two analysed samples, and their satDNA landscapes exhibit a consistent overall pattern. Notably, the sharp peaks observed at low K2P values suggest recent expansions of specific satDNA families located in the heterochromatic regions. In contrast, euchromatic satDNA families display broader peaks at higher divergence values, indicating older origins and dispersed genomic locations that hinder strong homogenisation. The main differences between the samples from Vietnam and China are due to two main satDNA families, TrubSat001‐166 and TrubSat002‐9. TrubSat002‐9 corresponds to the (GATA)_n_ repeat and its variants. Previous studies on other triatomines have shown that these repeats accumulate in the heterochromatin of the Y chromosome in all species of the Triatomini tribe, including those from the genus *Triatoma* (Mora et al., 2023; Panzera et al., 2023). This localisation may explain the differences in the percentage of this satDNA in the genome of the Vietnam and China samples, 3.77% and 0.02%, respectively. FISH analysis has demonstrated that in the Vietnam individual, this satDNA also accumulates exclusively in the Y chromosome (Figure 4d). Since the China sample corresponds to a female, which lacks a Y chromosome, it is logical that the amount of this satDNA is significantly lower. The differences observed for the TrubSat001‐166 family, 20.43% in the Vietnam genome and 5.48% in the China genome, cannot be explained by the different sexes of the samples. The genomic data from the female sample from China were retrieved directly from GenBank, so we cannot cytogenetically determine the localisation of this satDNA in that population. In the Vietnam male, this satDNA is located in the heterochromatin of the autosomes and is absent in the sex chromosomes (Figure 4c). Therefore, the amount of this satDNA should be similar in both sexes. The most plausible explanation for these differences is the presence of intraspecific polymorphism in the amount of autosomal heterochromatin between the two populations, as has been observed in other triatomines, such as *T. infestans* (Pita, Panzera, Sánchez, et al., 2017). Notwithstanding, several insect species have been reported to exhibit intraspecies variability in terms of satDNA abundance, i.e., the Heteroptera species *Euschistus heros* (Hickmann et al., 2023); two beetle species, *Euchroma gigantea*, Buprestidae (Félix et al., 2024) and *Rhynchophorus ferrugineus*, Curculionidae (Montiel et al., 2022); the Lepidoptera species *Spodoptera frugiperda* (Haq et al., 2022). Notably, within the *Drosophila simulans* species clade, bursts of amplifications were observed in euchromatic satDNA families, resembling a changing dynamic similar to that of TEs (Sproul et al., 2020). Similarly, in the Heteroptera species *Oxycarenus hyalinipennis*, highly abundant satDNA families were located within euchromatic regions, hence probably had undergone cycles of expansion and dispersion (Cabral‐de‐Mello et al., 2023). The mechanisms behind this variability between individuals could include unequal crossing‐over, ectopic recombination, transposition events, gene conversion, and rolling circle replication, as has been largely proposed for satDNA evolution (Belyayev et al., 2020; Charlesworth et al., 1994; Dover, 2002; Garrido‐Ramos, 2017; Plohl et al., 2012; Ugarković & Plohl, 2002).

Previous studies revealed a clear divergence in repetitive DNA composition between North and South American Triatomini lineages differing in the content of autosomal heterochromatic regions (Mora et al., 2023; Pita, Panzera, Mora, et al., 2017). Here, the satDNA study in *T. rubrofasciata* clearly showed its importance in genomic differentiation in North American species, similar to what has been described in South American species. For instance, the chromosomal sex systems vary significantly. North American species predominantly display multiple sex chromosome systems, such as X_1_X_2_Y or X_1_X_2_X_3_Y, whereas South American species mainly exhibit the XY system (reviewed in Panzera et al., 2021).

The differences in abundance of distinct families of satDNAs and the conservation of some of them between Triatominae species align with the ‘library hypothesis’, which proposes that a given ancestral genome harbours a pool of satDNA families that are independently amplified or lost across species during evolution (Fry & Salser, 1977). Before this work, the ‘library hypothesis’ was tested on a smaller evolutionary timescale through the comparison of satellitomes of two sister species of the South American lineage, *T. infestans* and *T. delpontei*, revealing that they share most of their satDNA families (Mora et al., 2023). As expected, due to the greater evolutionary distance, fewer satDNA families are shared between North and South American lineages compared with those observed within the South American lineage. The number of shared satDNA families is further reduced when comparing Triatoma species with *R. prolixus*. Specifically, nine satDNA families are present in the genomes of the three analysed *Triatoma* species. Among these, only three are also found in the genome of *R. prolixus* (Figure 2). The consensus sequences of these satDNA families exhibit a high degree of conservation across different species, with percent identity exceeding 78.50% in all cases and being identical for the shorter satDNA families (Figure S4). This conservation among species indicates that the origin of these satDNA families predates species diversification in Triatominae, which have diverged recently (∼32 Ma) (Hwang & Weirauch, 2012). This evolutionary stability could imply functional relevance, warranting further investigation. In other organisms, non‐coding RNAs derived from satDNA have been implicated in critical cellular processes, including gene expression regulation, chromosomal architecture maintenance, and cellular stress responses (Biscotti et al., 2015; Pezer & Ugarković, 2008). For instance, in *Drosophila*, satellite‐derived RNAs influence nuclear organisation and genomic stability (Ferree & Barbash, 2009; Jagannathan et al., 2018).

**FIGURE 5:**
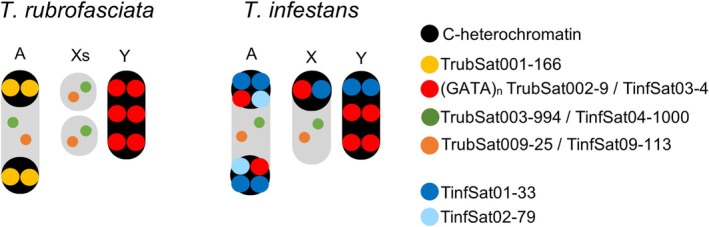
Schematic representation of comparative karyotype evolution between *Triatoma rubrofasciata* and *Triatoma infestans*. Shared satDNA families are shown with species‐specific chromosomal locations. Euchromatic regions are depicted in grey, while heterochromatic regions are indicated in black. Chromosome location of the satDNAs in *T. infestans* were obtained in a previous study (Pita, Panzera, Mora, et al., 2017).

Interestingly, the differentiation and patterns of amplification of some satDNA families among Triatominae are strongly linked to heterochromatin expansion, mainly on autosomes (Figure 5). This is evident from the comparison of the chromosomal location of certain satDNAs across species, revealing that some of them are located in the euchromatin and present in low abundance in one species, whereas in others, they occur in high abundance within heterochromatin blocks, and vice versa. While conserved repetitive sequences are typically confined to the Y chromosome, each species' autosomal C‐heterochromatin is composed of distinct satDNA families (Panzera et al., 2023; Pita et al., 2014; Pita, Panzera, Sánchez, et al., 2017). An example of this dynamism is the satDNA TrubSat001‐166, the major satDNA family in *T. rubrofasciata*, which is present in *T. infestans* and absent in *T. delpontei*. TrubSat001‐166 forms the C‐heterochromatic blocks in *T. rubrofasciata*, representing 20.43% of the genome in Vietnam and 5.48% in China. However, the same satDNA in *T. infestans* (TinfSat075‐167) is present at a very low proportion in the genome, around 0.005% in the Andean group, and was not found in the non‐Andean group. In a similar way, the satDNAs that mainly constitute the heterochromatin in *T. infestans* or *T. delpontei*, have not been found in any of the analysed samples of *T. rubrofasciata*. A special case would be the GATA repeats and its variants. In *T. infestans* and *T. delpontei*, GATA sequences, in addition to the Y chromosome, have also been amplified in the heterochromatin of the autosomes and the X chromosome (Mora et al., 2023; Pita, Panzera, Mora, et al., 2017), but not in *T. rubrofasciata*. Subtelomeric regions in *Triatoma* seem to facilitate the amplification and subsequent dispersal of satDNA families through ectopic recombination, as proposed in earlier studies (Mora et al., 2023). This mechanism likely contributes to the independent formation of C‐heterochromatin within different lineages, potentially influencing chromosomal architecture. Among other insects, similar patterns for satDNA library evolution are also observed, such as in *Adalia* beetles, with a highly repetitive satDNA that forms the heterochromatin in *A. bipunctata* but is absent in *A. decempunctata*, underscoring the dynamic evolution of satDNA even within closely related species (Mora et al., 2024). The processes of differential amplification among satDNA families, or their stochastic loss, along with the birth and expansion of new satDNAs, would lead to the differentiation of satellitomes among related species, as has been extensively analysed among 37 species of the *Drosophila* genus (de Lima & Ruiz‐Ruano, 2022).

In this study, we expanded the knowledge about satDNA library evolution in Triatomini as *T. rubrofasciata* belongs to the North American lineage, shedding light on the identification of several traits related to karyotypic and genomic evolution. Our findings revealed that satDNA remains the most abundant component of the repeatome in *T. rubrofasciata*, with a similar repetitive DNA fraction across genomes. This is also a common feature in other South American Triatomini lineages (Mora et al., 2023; Pita, Panzera, Mora, et al., 2017). In contrast, the repeatome analysis in the genus *Rhodnius*, which belongs to the sister tribe Rhodniini, revealed that TEs are the most abundant repetitive DNAs on its genomes (Castro et al., 2020; Fernandez‐Medina et al., 2016; Montiel et al., 2021). Our study broadens the knowledge of repetitive DNAs in Triatominae genomes, not only shaping genome architecture but also playing a pivotal role in karyotypic evolution. It is plausible to hypothesise that heterochromatin formation occurred independently multiple times or experienced intense turnover within the Triatomini tribe, as supported by our findings on the sequence components of C‐heterochromatin. Future studies could explore whether this hypothesis holds true across species from different subcomplexes of both lineages. These findings emphasise the need for further research into these mechanisms, which may also shed light on lineages differentiation and speciation processes within this diverse genus.

## AUTHOR CONTRIBUTIONS

**Sebastián Pita:** Conceptualization; investigation; writing – original draft; writing – review and editing. **Pablo Mora:** Conceptualization; investigation; writing – original draft; writing – review and editing. **José M. Rico‐Porras:** Investigation; writing – review and editing. **Diogo C. Cabral‐de‐Mello:** Investigation; writing – review and editing. **Francisco J. Ruiz‐Ruano:** Investigation; writing – review and editing. **Teresa Palomeque:** Investigation; writing – review and editing; funding acquisition. **Ho Viet Hieu:** Investigation; resources; writing – review and editing. **Francisco Panzera:** Investigation; writing – review and editing. **Pedro Lorite:** Conceptualization; investigation; writing – original draft; writing – review and editing; funding acquisition.

## CONFLICT OF INTEREST STATEMENT

The authors declare no conflict of interests.

## Supporting information


**Figure S1.** *Triatoma rubrofasciata* satDNA landscapes (abundance as a percentage vs. K2P divergence as a percentage) for the Vietnam and China samples.

**Figure S2.** *Triatoma rubrofasciata* individual satDNA landscapes (abundance as a percentage vs. K2P divergence as a percentage) for the Vietnam sample.

**Figure S3.** *Triatoma rubrofasciata* individual satDNA landscapes (abundance as a percentage vs. K2P divergence as a percentage) for the China sample.

**Figure S4.** Alignments of the shared satDNAs among *Triatoma rubrofasciata* and others Triatomini.

**Table S1.** General features of the satDNA families found in *Triatoma rubrofasciata*.

**Table S2.** Abundance and divergence of the satDNAs of *Triatoma rubrofasciata* satellitome in the Vietnam and China samples. ND = non‐detected.

## Data Availability

The data that support the findings of this study are openly available in GenBank at https://www.ncbi.nlm.nih.gov/genbank/ (accession number SAMN48410004, and PV637482‐PV637621) https://www.ncbi.nlm.nih.gov/biosample/SAMN48410004/, https://www.ncbi.nlm.nih.gov/nuccore/PV637482.
