# Supplementary material for: A new piece in the repeatome puzzle of Triatominae bugs: The analysis of Triatoma rubrofasciata reveals the role of satellite DNAs in the karyotypic evolution of distinct lineages
Source: Insect Mol Biol. 2025 Jun 27;34(6):917–28. doi: 10.1111/imb.13013 (PMC12604443; doi:10.1111/imb.13013)

Vietman sample

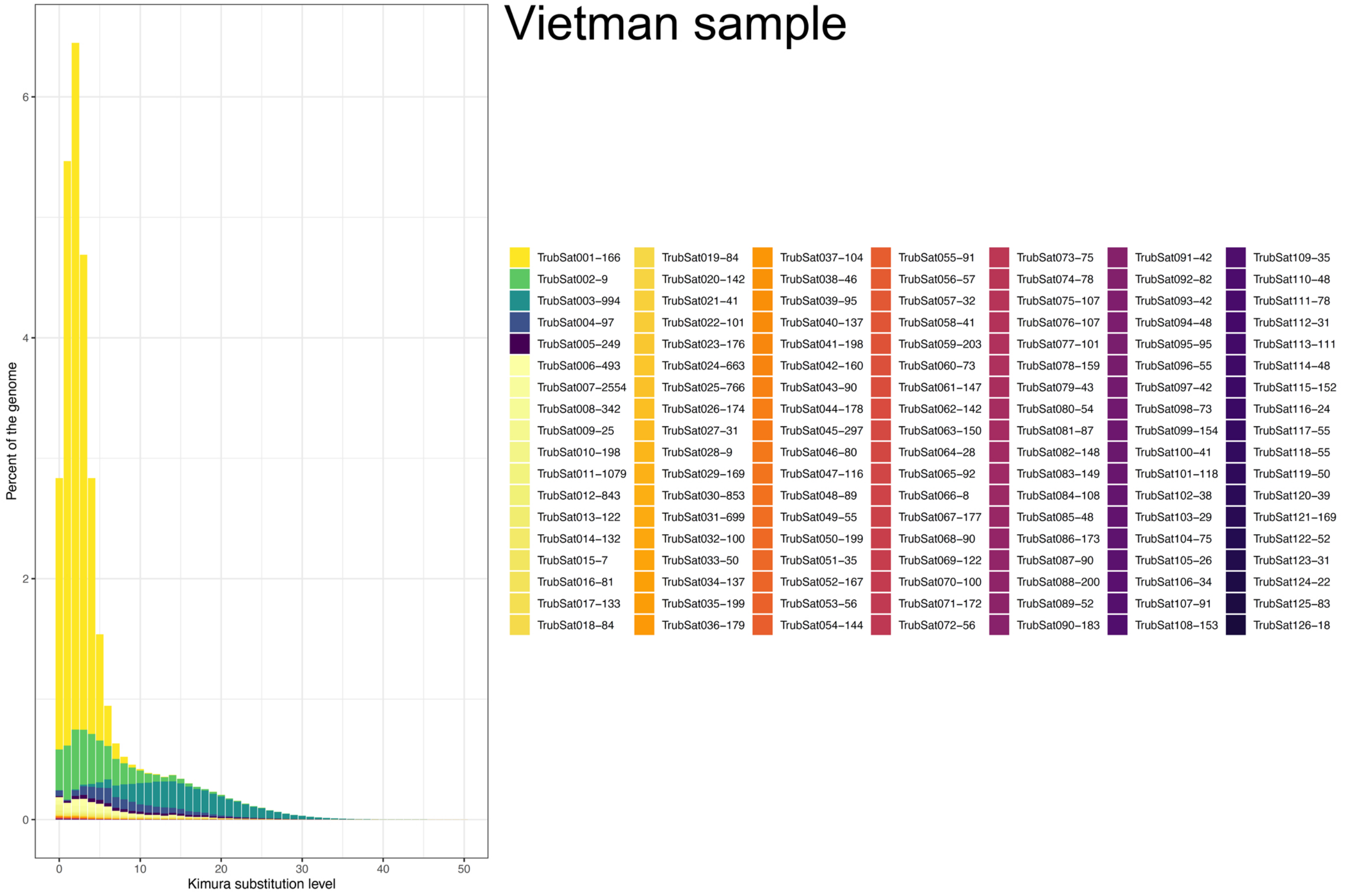

China sample

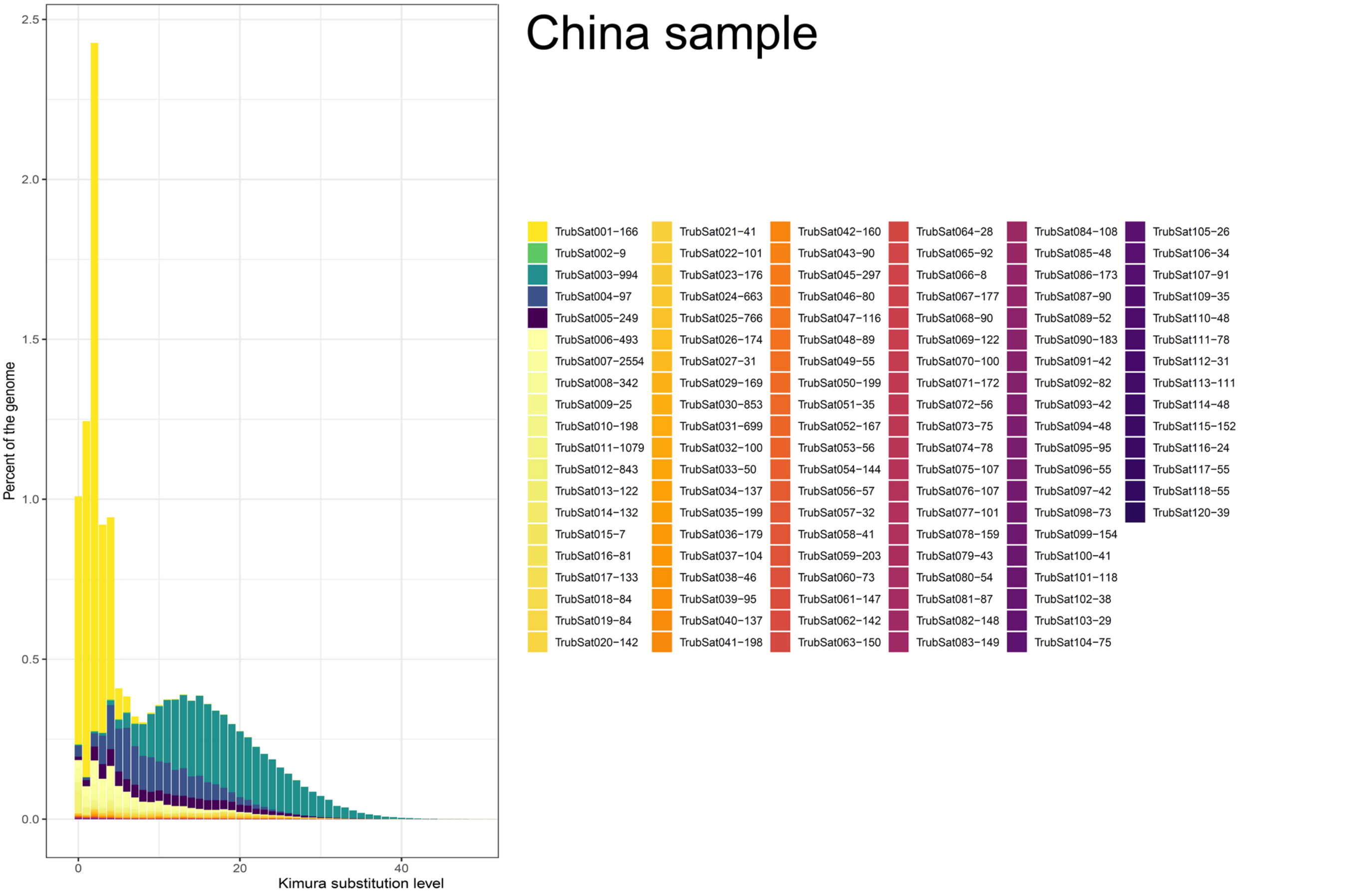

Supplement: Supplementary file 1 — Figure S1. Triatoma rubrofasciata satDNA landscapes (abundance as a percentage vs. K2P divergence as a percentage) for the Vietnam and China samples. [file IMB-34-917-s003.pdf]
