# Supplementary material for: A new piece in the repeatome puzzle of Triatominae bugs: The analysis of Triatoma rubrofasciata reveals the role of satellite DNAs in the karyotypic evolution of distinct lineages
Source: Insect Mol Biol. 2025 Jun 27;34(6):917–28. doi: 10.1111/imb.13013 (PMC12604443; doi:10.1111/imb.13013)
Supplement: Supplementary file 2 — Figure S2. Triatoma rubrofasciata individual satDNA landscapes (abundance as a percentage vs. K2P divergence as a percentage) for the Vietnam sample. [file IMB-34-917-s006.pdf]

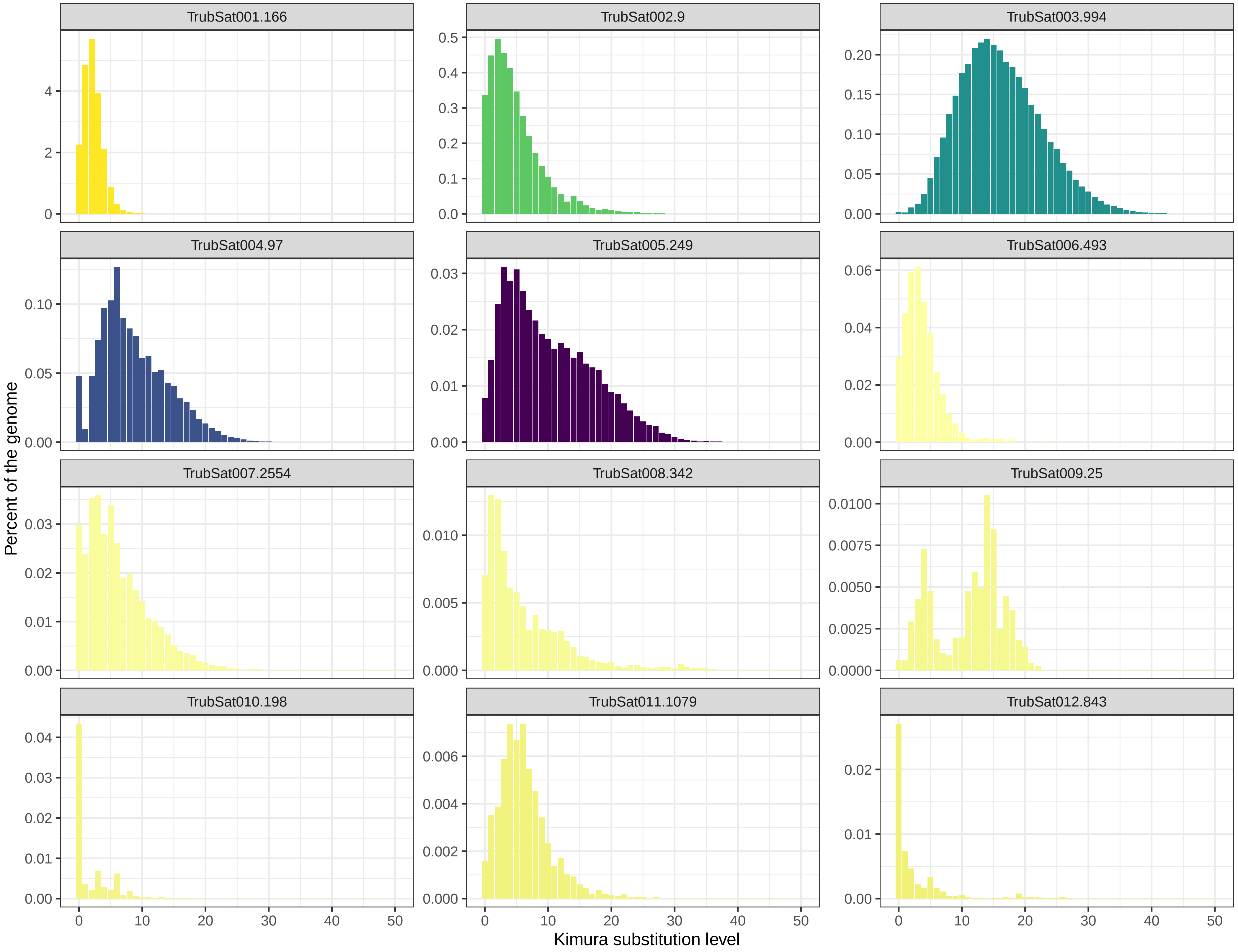
TrubSat001.166
TrubSat002.9
TrubSat003.994
TrubSat004.97
TrubSat005.249
TrubSat006.493
TrubSat007.2554
TrubSat008.342
TrubSat009.25
TrubSat010.198
TrubSat011.1079
TrubSat012.843
Percent of the genome
Kimura substitution level
0
10
20
30
40
50

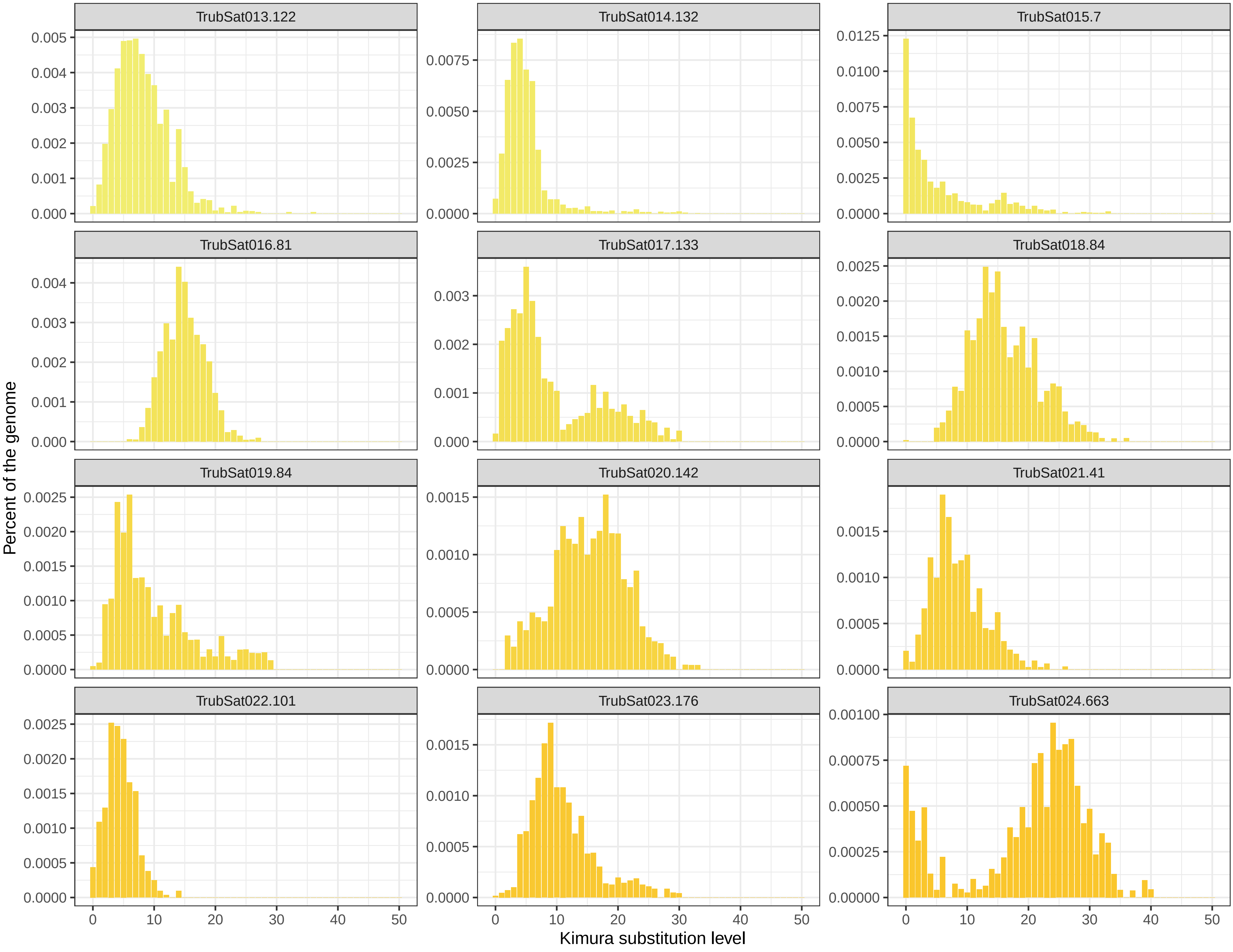

TrubSat013.122
TrubSat014.132
TrubSat015.7
TrubSat016.81
TrubSat017.133
TrubSat018.84
TrubSat019.84
TrubSat020.142
TrubSat021.41
TrubSat022.101
TrubSat023.176
TrubSat024.663
Percent of the genome
Kimura substitution level

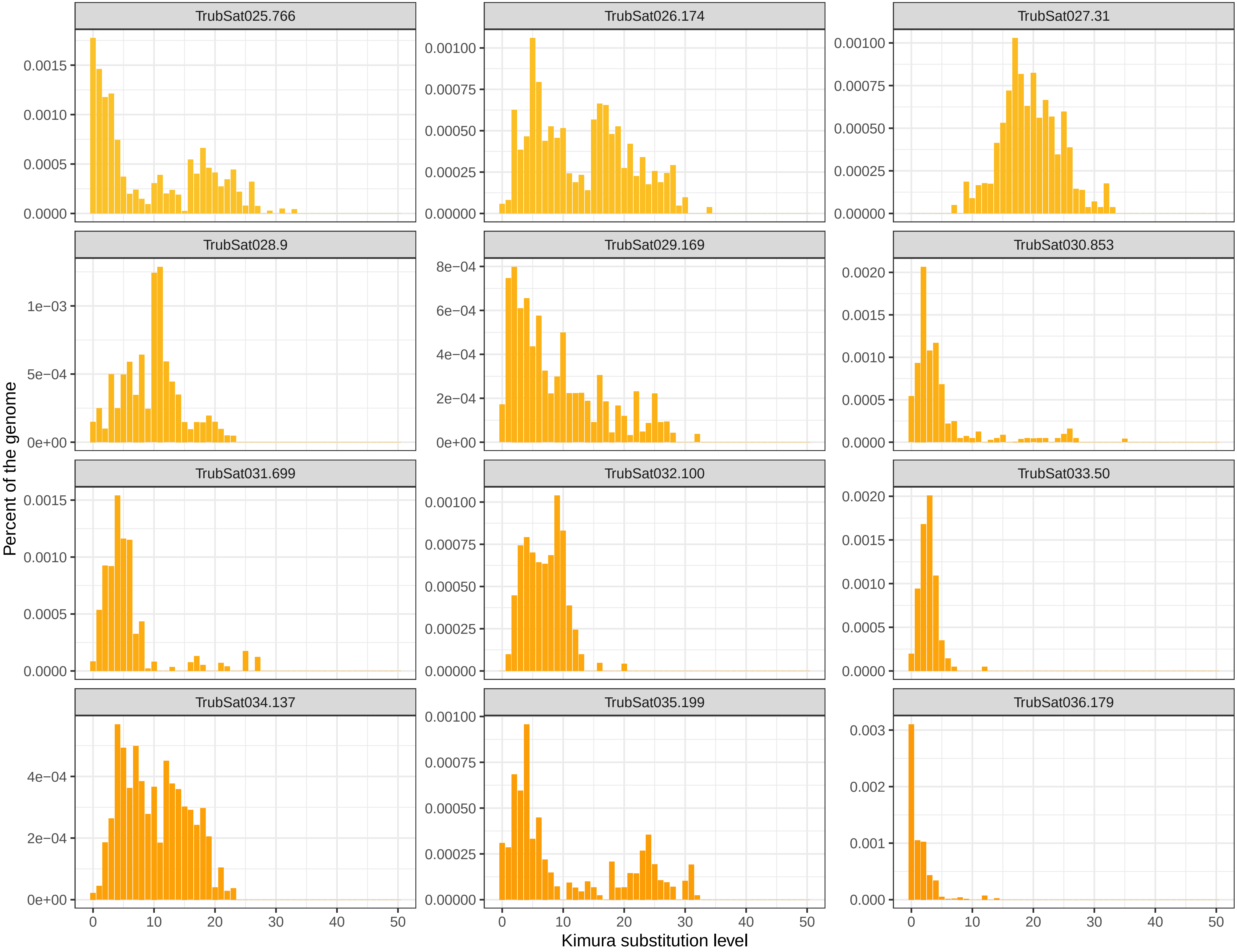
TrubSat025.766
TrubSat026.174
TrubSat027.31
TrubSat028.9
TrubSat029.169
TrubSat030.853
TrubSat031.699
TrubSat032.100
TrubSat033.50
TrubSat034.137
TrubSat035.199
TrubSat036.179
Percent of the genome
Kimura substitution level

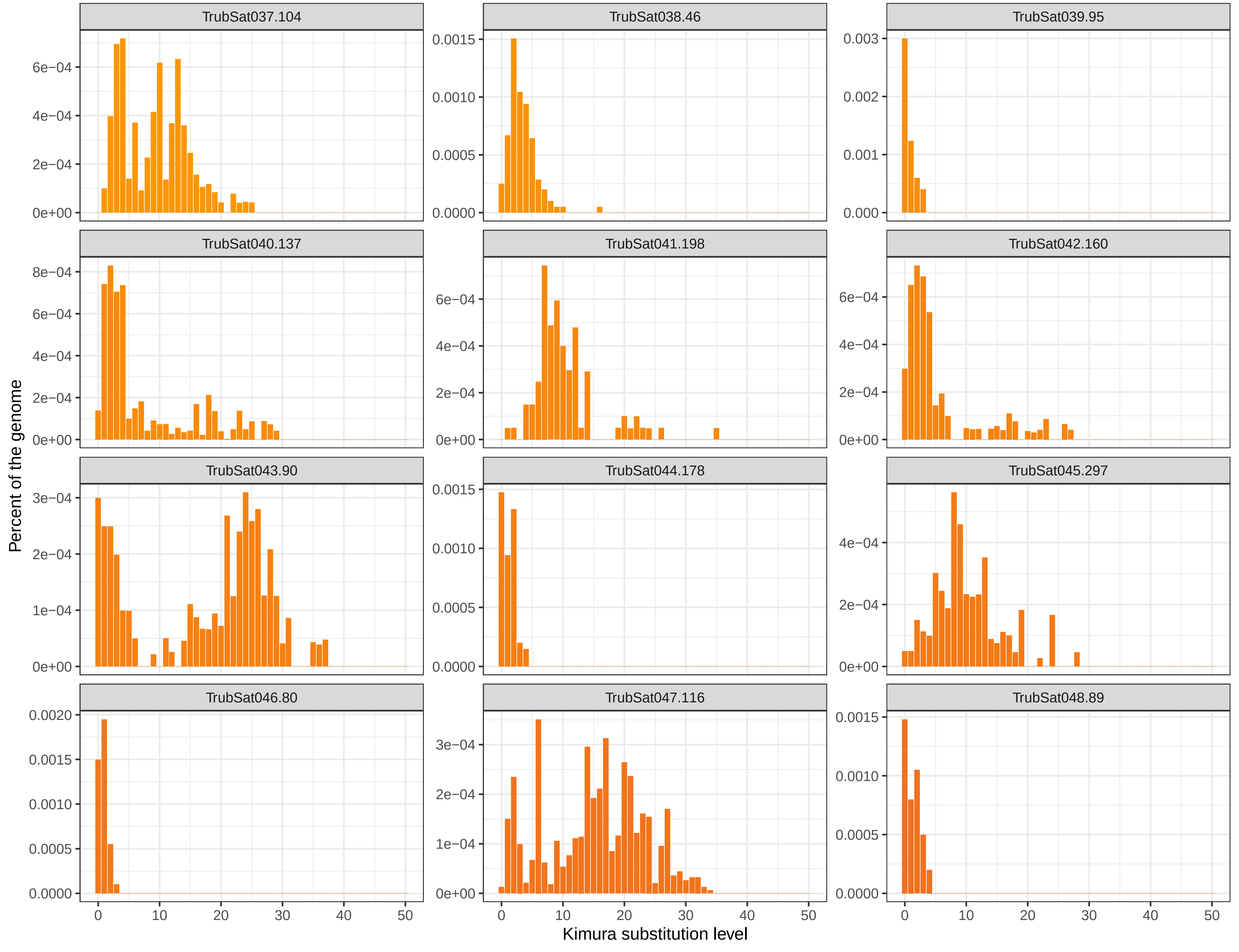
TrubSat037.104
TrubSat038.46
TrubSat039.95
TrubSat040.137
TrubSat041.198
TrubSat042.160
TrubSat043.90
TrubSat044.178
TrubSat045.297
TrubSat046.80
TrubSat047.116
TrubSat048.89
Percent of the genome
Kimura substitution level

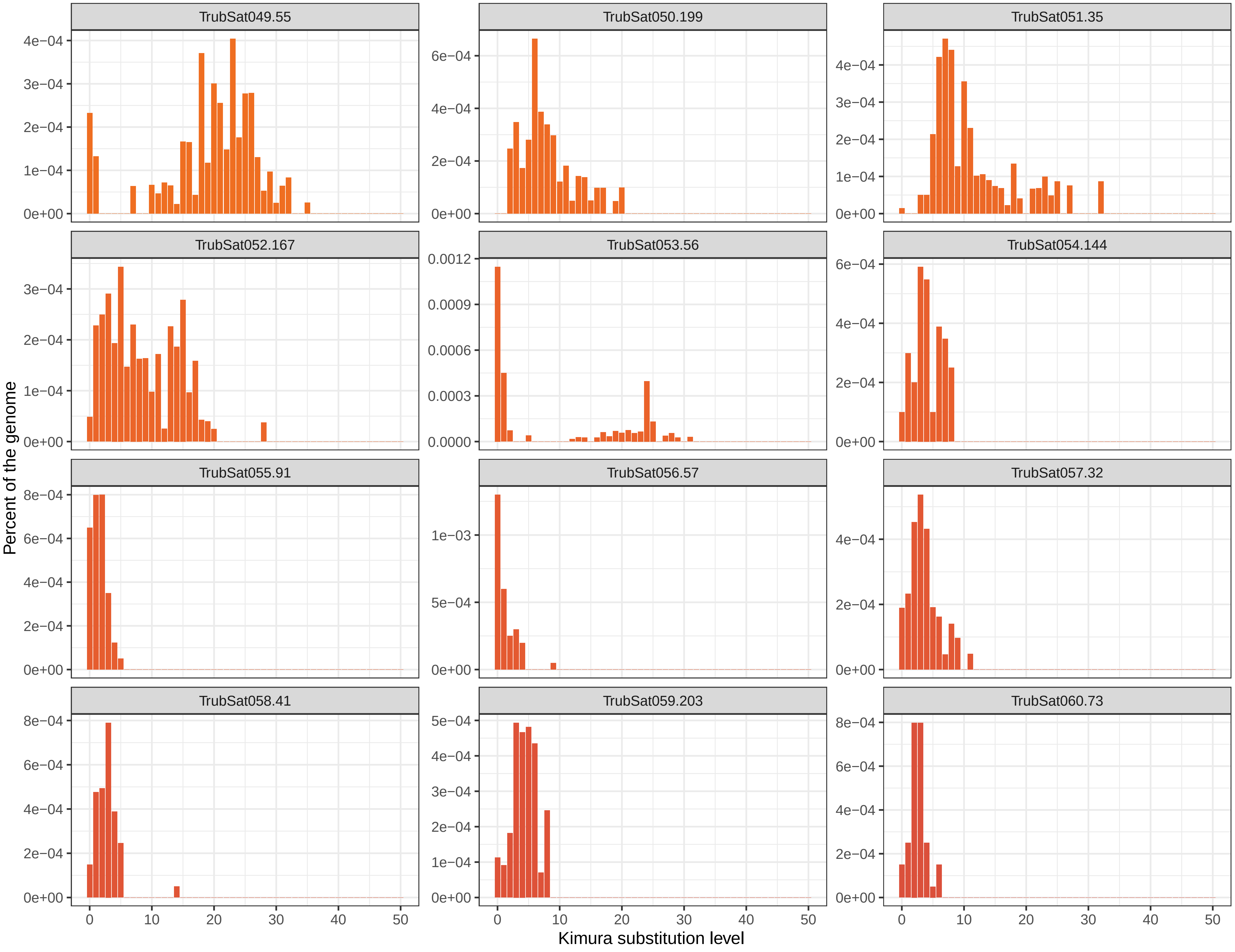
TrubSat049.55
TrubSat050.199
TrubSat051.35
TrubSat052.167
TrubSat053.56
TrubSat054.144
TrubSat055.91
TrubSat056.57
TrubSat057.32
TrubSat058.41
TrubSat059.203
TrubSat060.73
Percent of the genome
Kimura substitution level

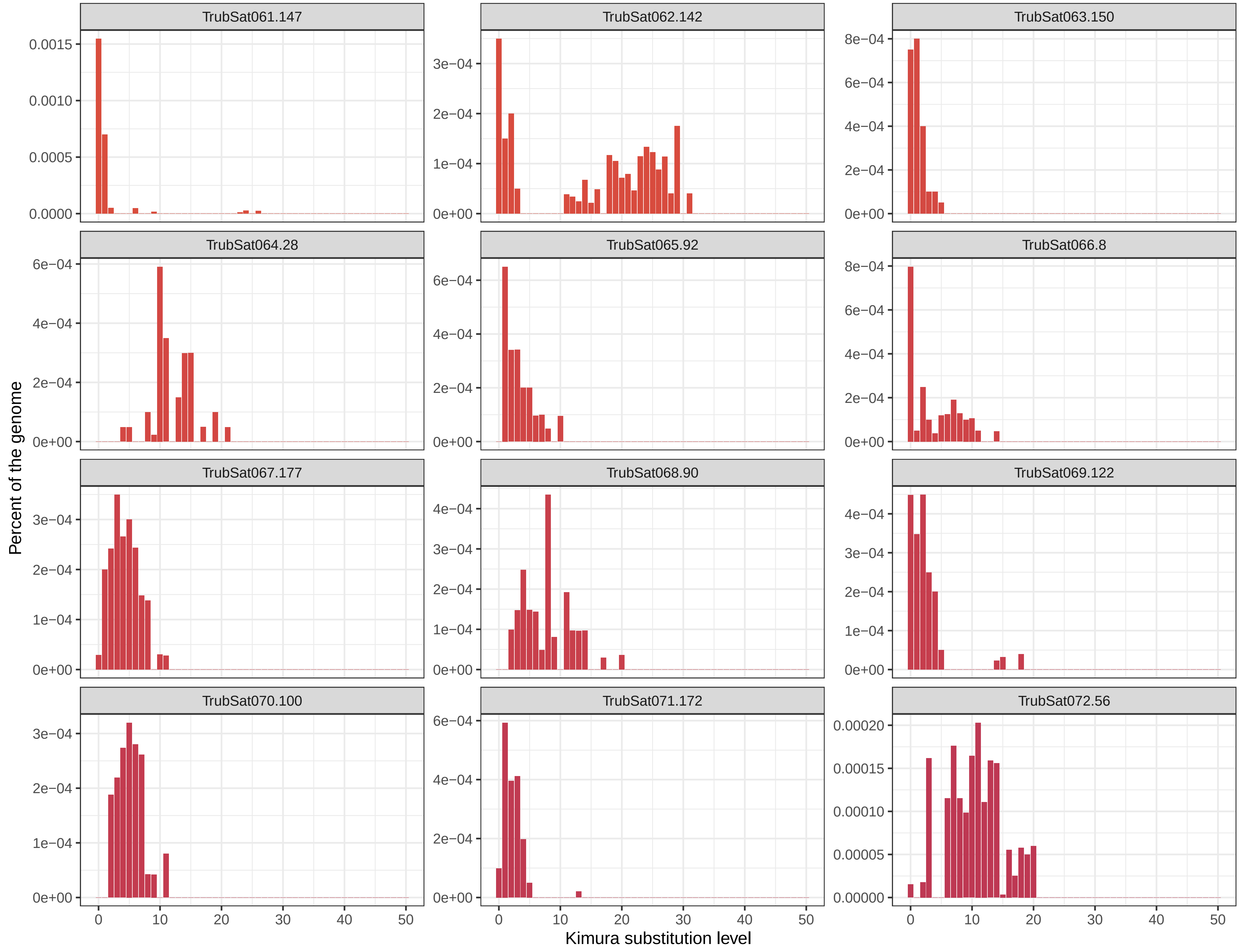
TrubSat061.147
TrubSat062.142
TrubSat063.150
TrubSat064.28
TrubSat065.92
TrubSat066.8
TrubSat067.177
TrubSat068.90
TrubSat069.122
TrubSat070.100
TrubSat071.172
TrubSat072.56
Percent of the genome
Kimura substitution level

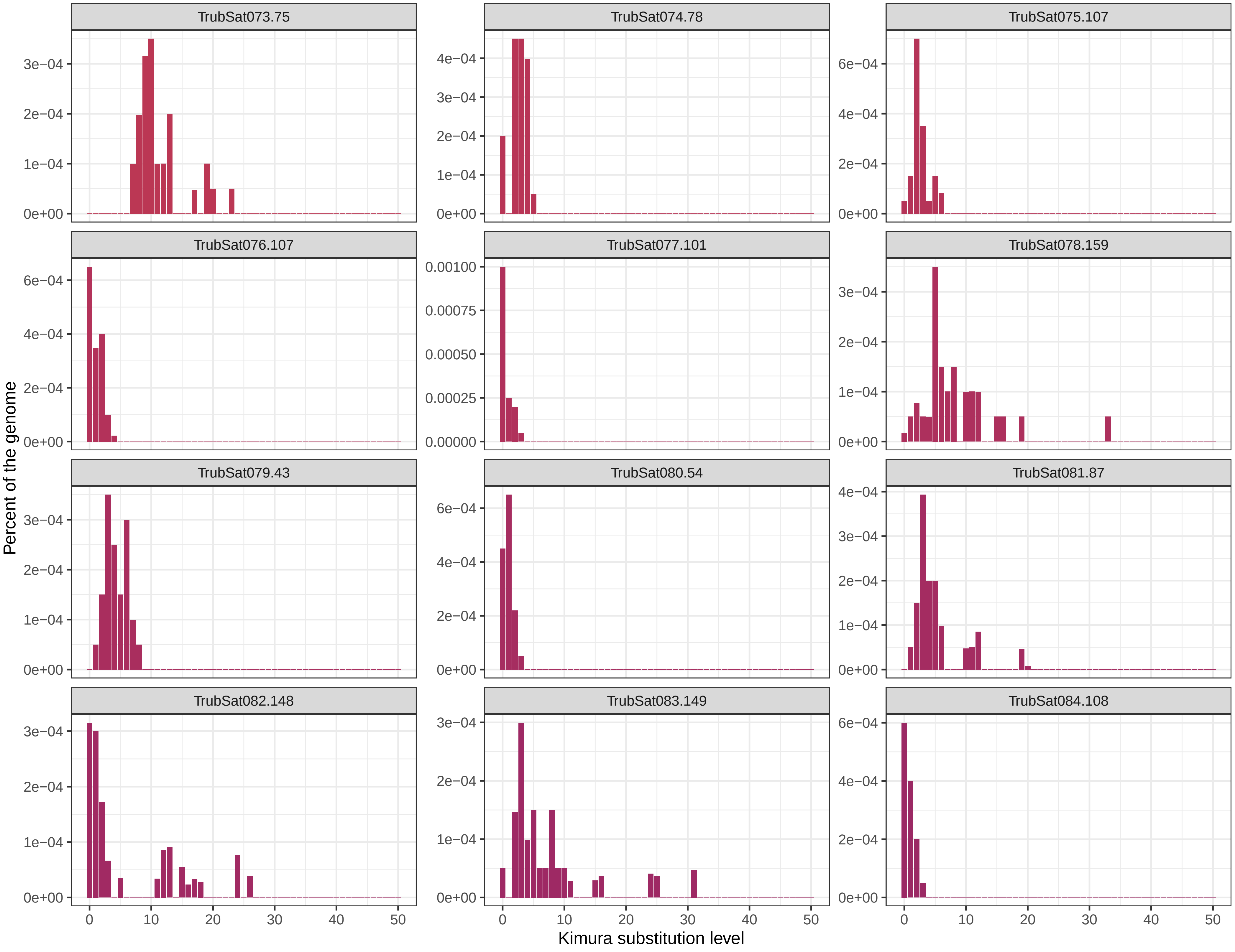
TrubSat073.75
TrubSat074.78
TrubSat075.107
TrubSat076.107
TrubSat077.101
TrubSat078.159
TrubSat079.43
TrubSat080.54
TrubSat081.87
TrubSat082.148
TrubSat083.149
TrubSat084.108
Percent of the genome
Kimura substitution level

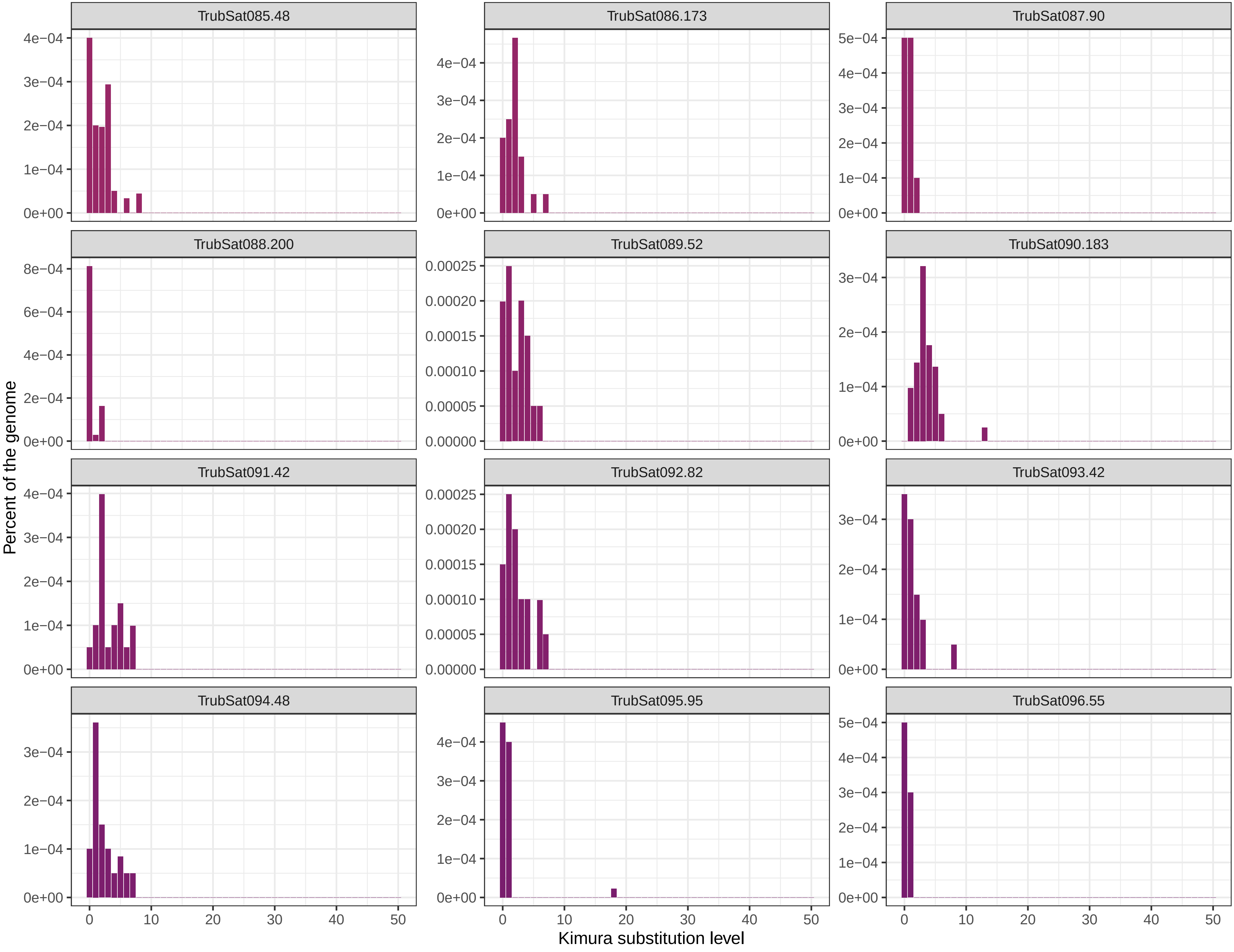

TrubSat085.48
TrubSat086.173
TrubSat087.90
TrubSat088.200
TrubSat089.52
TrubSat090.183
TrubSat091.42
TrubSat092.82
TrubSat093.42
TrubSat094.48
TrubSat095.95
TrubSat096.55
Percent of the genome
Kimura substitution level

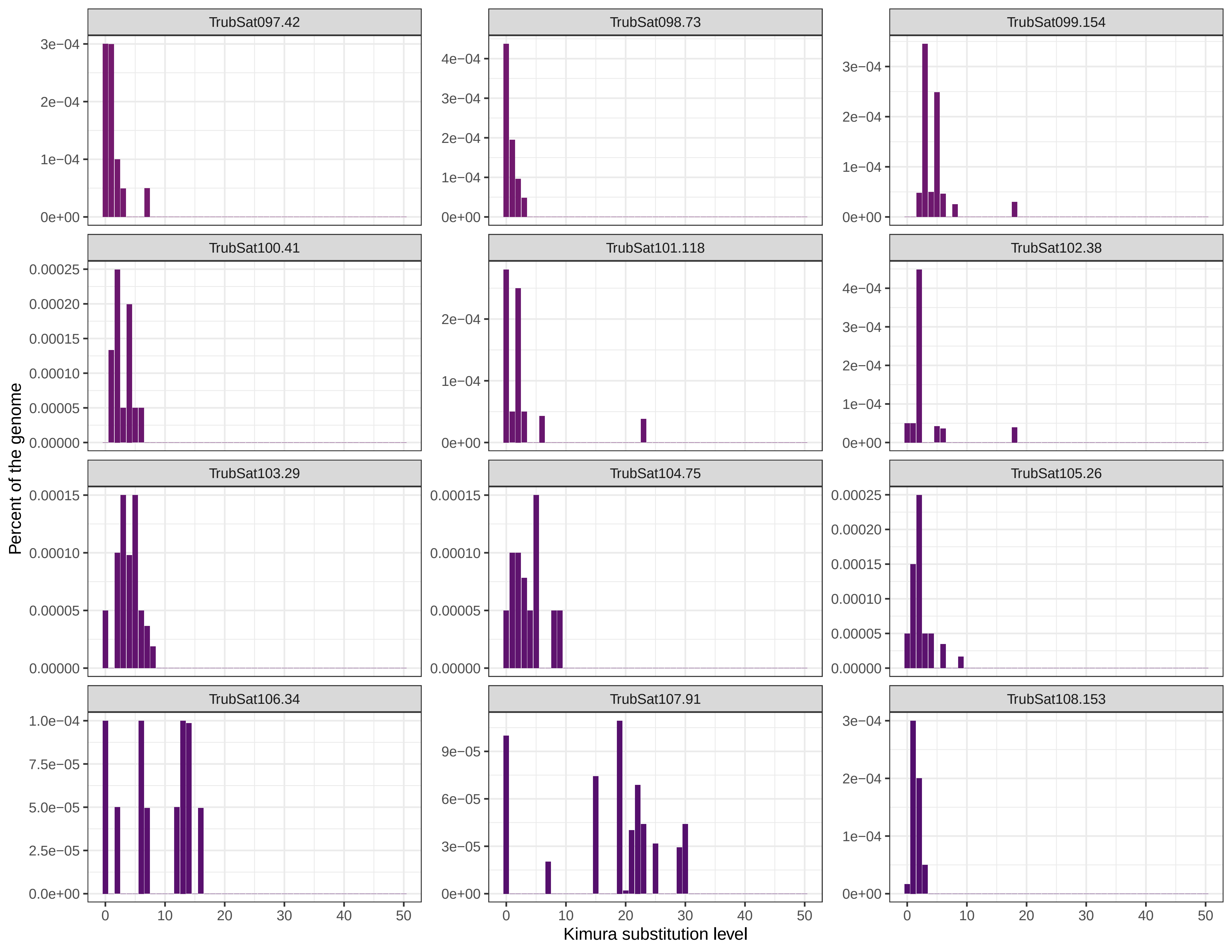

TrubSat097.42
TrubSat098.73
TrubSat099.154
TrubSat100.41
TrubSat101.118
TrubSat102.38
TrubSat103.29
TrubSat104.75
TrubSat105.26
TrubSat106.34
TrubSat107.91
TrubSat108.153
Percent of the genome
Kimura substitution level

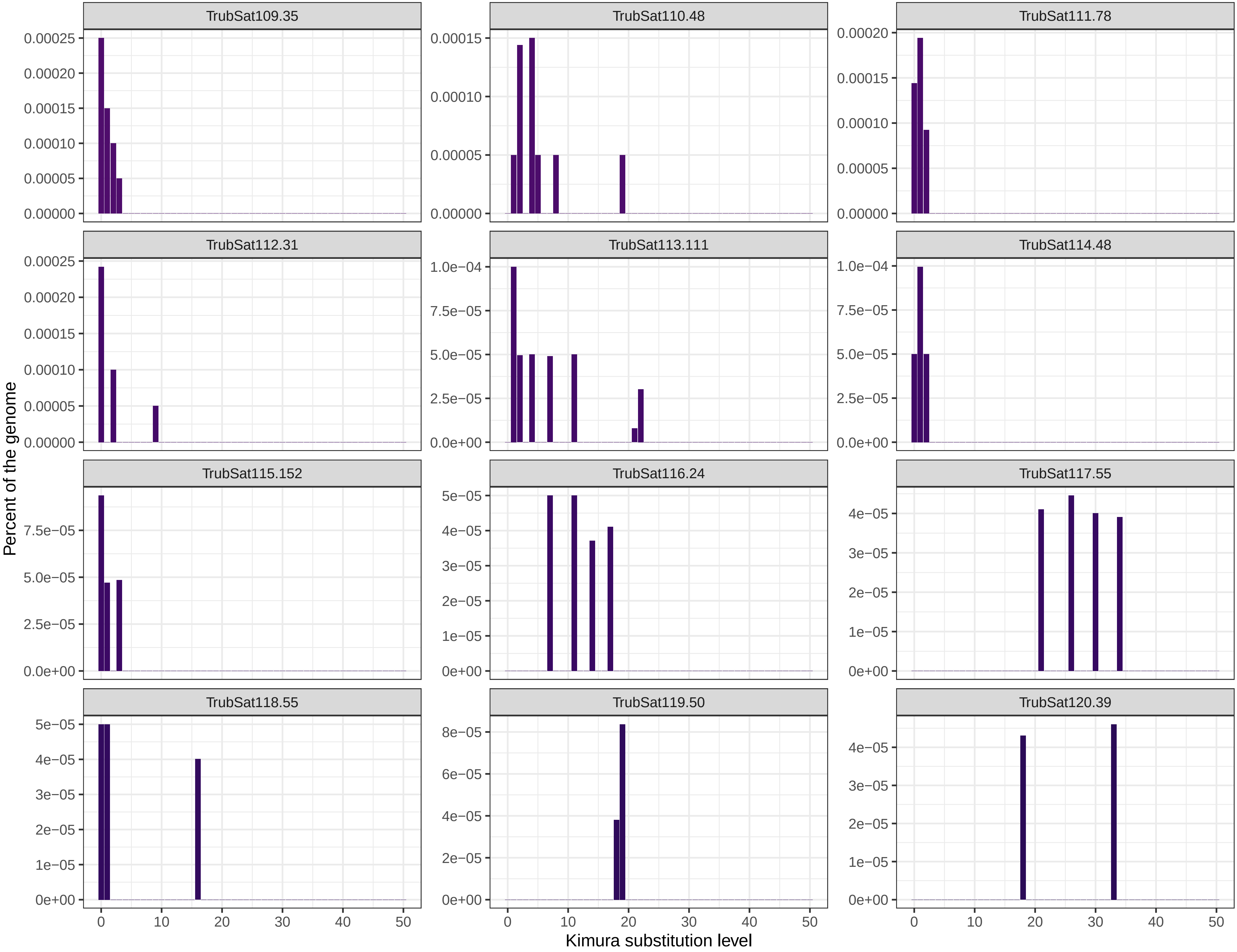
TrubSat109.35
TrubSat110.48
TrubSat111.78
TrubSat112.31
TrubSat113.111
TrubSat114.48
TrubSat115.152
TrubSat116.24
TrubSat117.55
TrubSat118.55
TrubSat119.50
TrubSat120.39
Percent of the genome
Kimura substitution level
0.00025
0.00020
0.00015
0.00010
0.00005
0.00000
1.0e−04
7.5e−05
5.0e−05
2.5e−05
0.0e+00
5e−05
4e−05
3e−05
2e−05
1e−05
0e+00
8e−05
6e−05
0
10
20
30
40
50

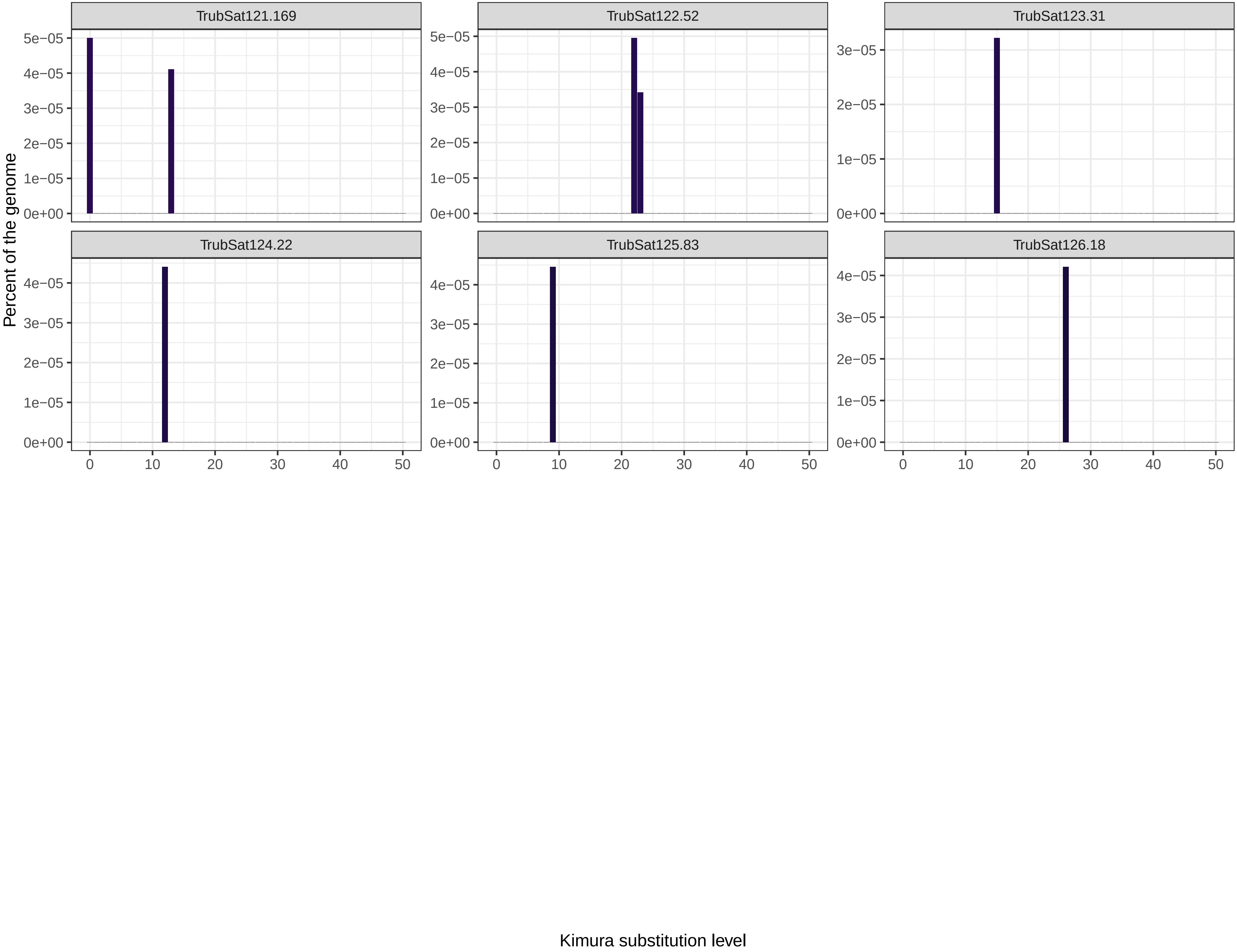
TrubSat121.169
TrubSat122.52
TrubSat123.31
TrubSat124.22
TrubSat125.83
TrubSat126.18
Percent of the genome
Kimura substitution level
