# Supplementary material for: A new piece in the repeatome puzzle of Triatominae bugs: The analysis of Triatoma rubrofasciata reveals the role of satellite DNAs in the karyotypic evolution of distinct lineages
Source: Insect Mol Biol. 2025 Jun 27;34(6):917–28. doi: 10.1111/imb.13013 (PMC12604443; doi:10.1111/imb.13013)
Supplement: Supplementary file 3 — Figure S3. Triatoma rubrofasciata individual satDNA landscapes (abundance as a percentage vs. K2P divergence as a percentage) for the China sample. [file IMB-34-917-s005.pdf]

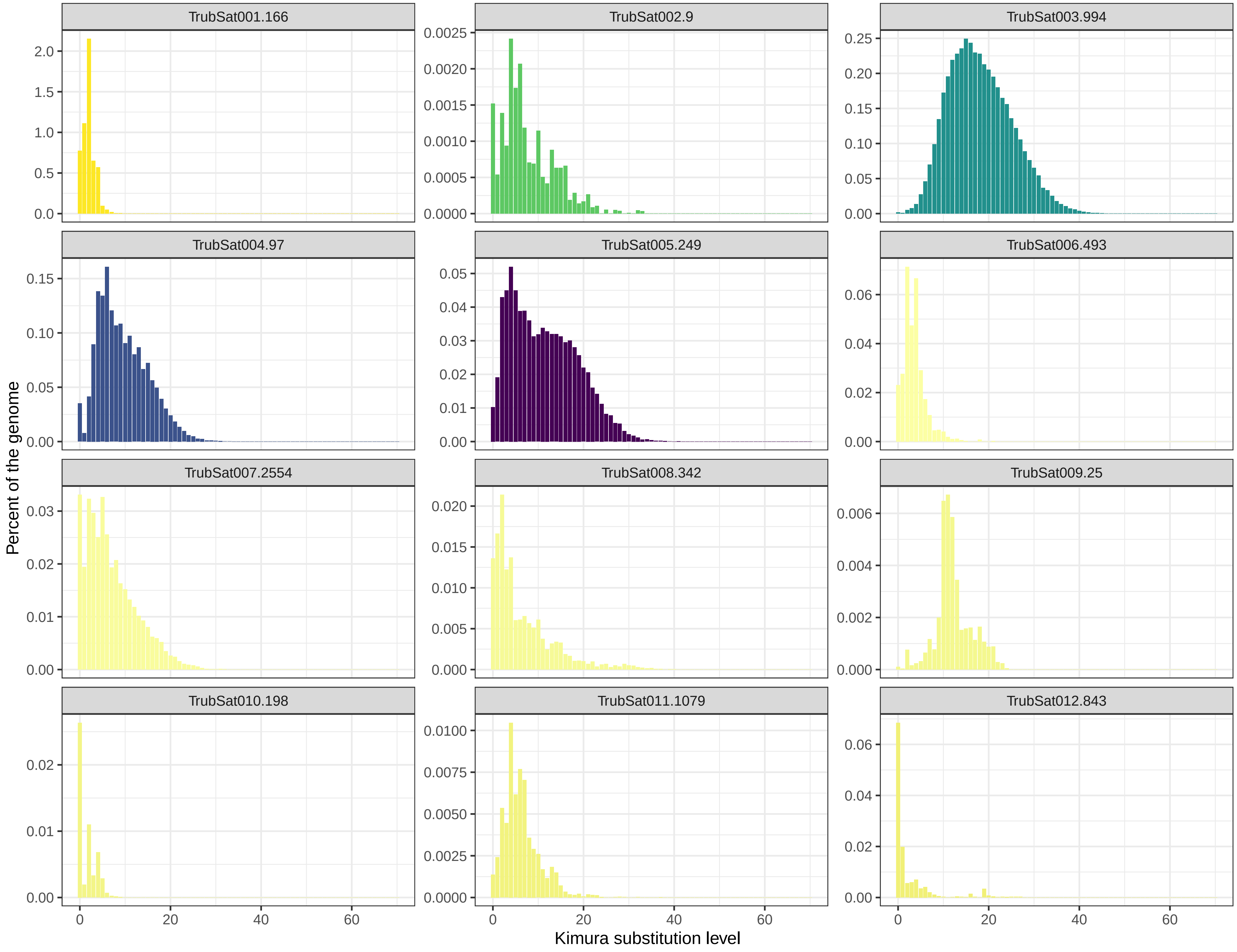
TrubSat001.166
TrubSat002.9
TrubSat003.994
TrubSat004.97
TrubSat005.249
TrubSat006.493
TrubSat007.2554
TrubSat008.342
TrubSat009.25
TrubSat010.198
TrubSat011.1079
TrubSat012.843
Percent of the genome
Kimura substitution level
0
20
40
60

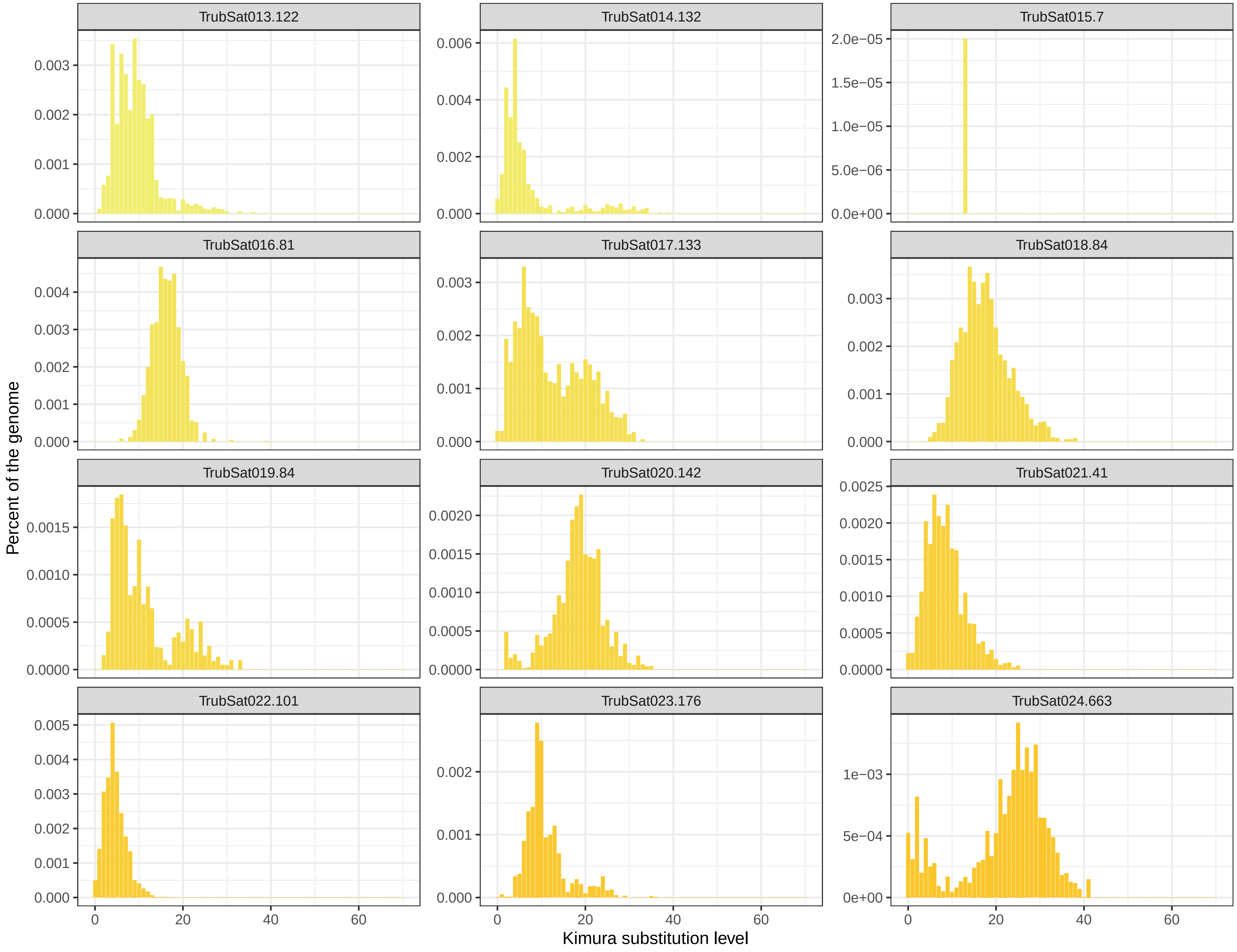

TrubSat013.122
TrubSat014.132
TrubSat015.7
TrubSat016.81
TrubSat017.133
TrubSat018.84
TrubSat019.84
TrubSat020.142
TrubSat021.41
TrubSat022.101
TrubSat023.176
TrubSat024.663
Percent of the genome
Kimura substitution level

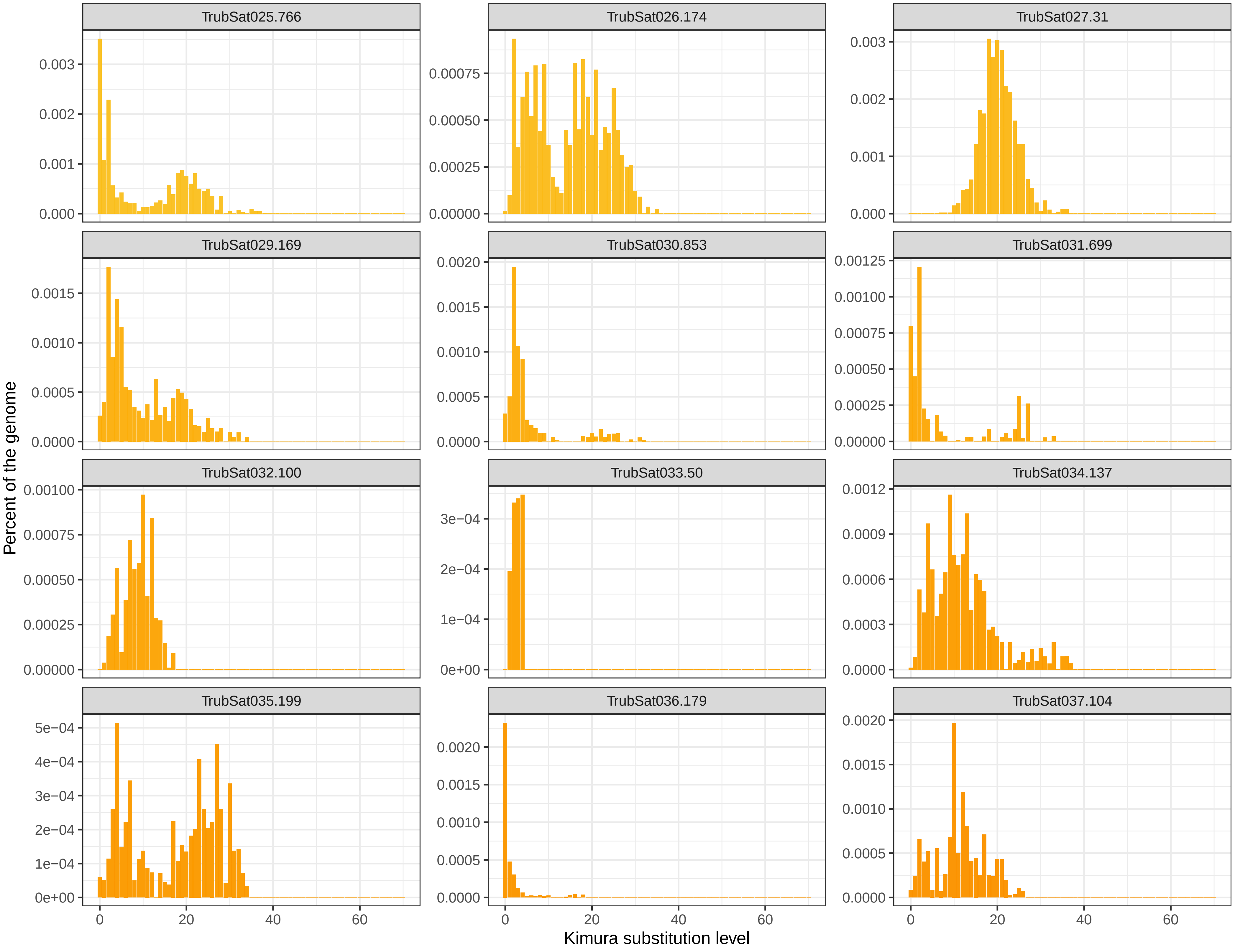
TrubSat025.766
TrubSat026.174
TrubSat027.31
TrubSat029.169
TrubSat030.853
TrubSat031.699
TrubSat032.100
TrubSat033.50
TrubSat034.137
TrubSat035.199
TrubSat036.179
TrubSat037.104
Percent of the genome
Kimura substitution level
0
20
40
60

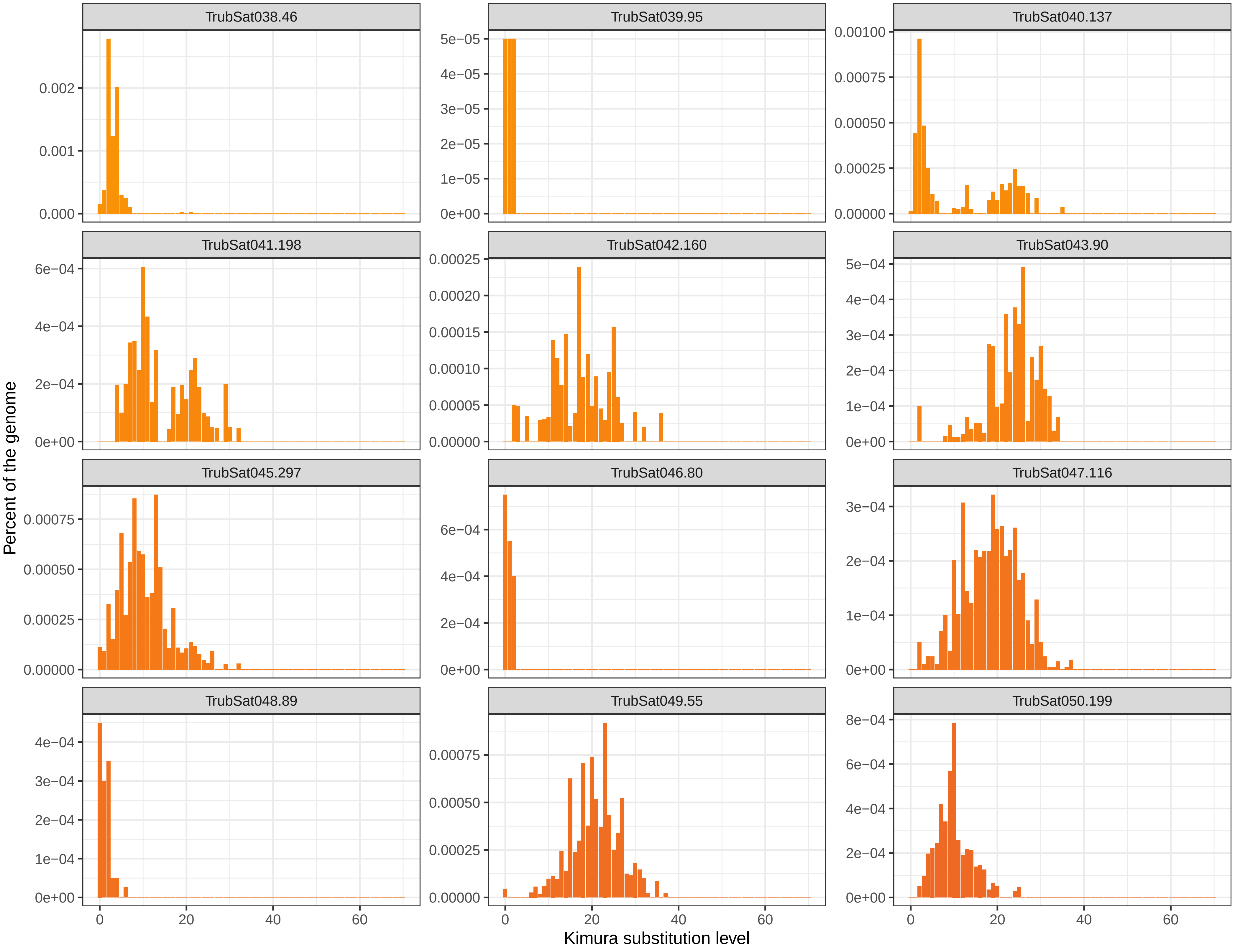
TrubSat038.46
TrubSat039.95
TrubSat040.137
TrubSat041.198
TrubSat042.160
TrubSat043.90
TrubSat045.297
TrubSat046.80
TrubSat047.116
TrubSat048.89
TrubSat049.55
TrubSat050.199
Percent of the genome
Kimura substitution level
0
20
40
60

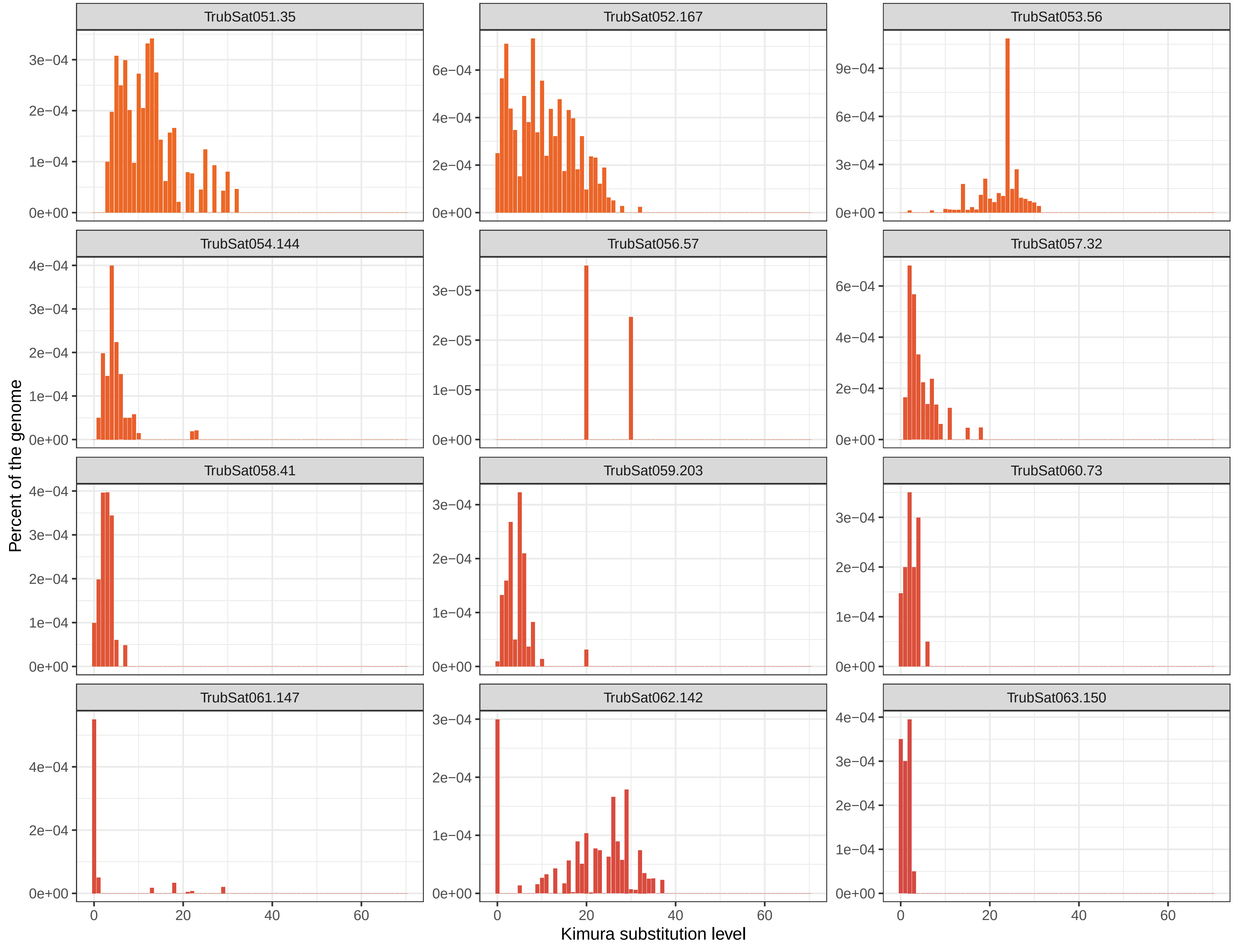

TrubSat051.35
TrubSat052.167
TrubSat053.56
TrubSat054.144
TrubSat056.57
TrubSat057.32
TrubSat058.41
TrubSat059.203
TrubSat060.73
TrubSat061.147
TrubSat062.142
TrubSat063.150
Percent of the genome
Kimura substitution level

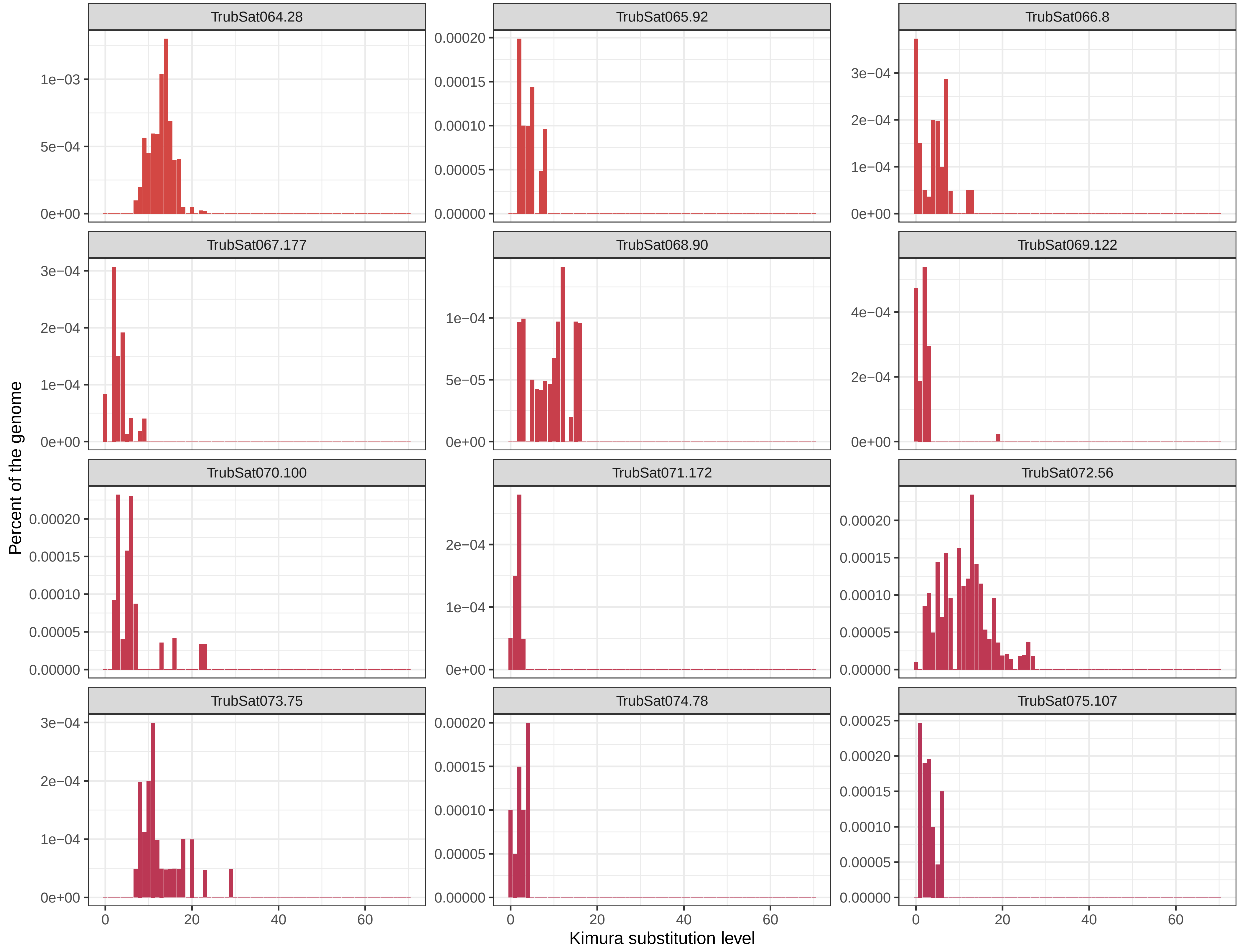
TrubSat064.28
TrubSat065.92
TrubSat066.8
TrubSat067.177
TrubSat068.90
TrubSat069.122
TrubSat070.100
TrubSat071.172
TrubSat072.56
TrubSat073.75
TrubSat074.78
TrubSat075.107
Percent of the genome
Kimura substitution level

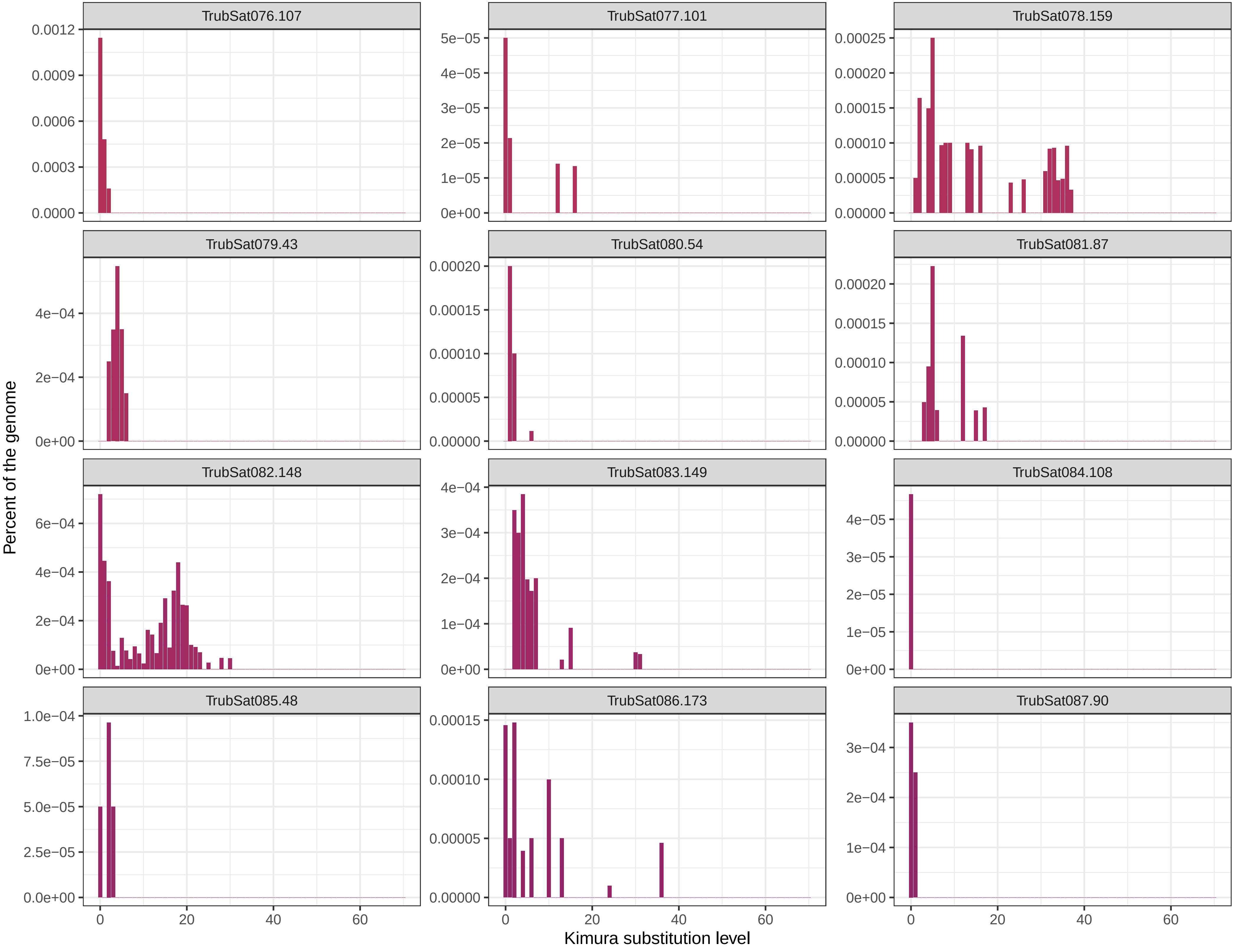

TrubSat076.107
TrubSat077.101
TrubSat078.159
TrubSat079.43
TrubSat080.54
TrubSat081.87
TrubSat082.148
TrubSat083.149
TrubSat084.108
TrubSat085.48
TrubSat086.173
TrubSat087.90
Percent of the genome
Kimura substitution level

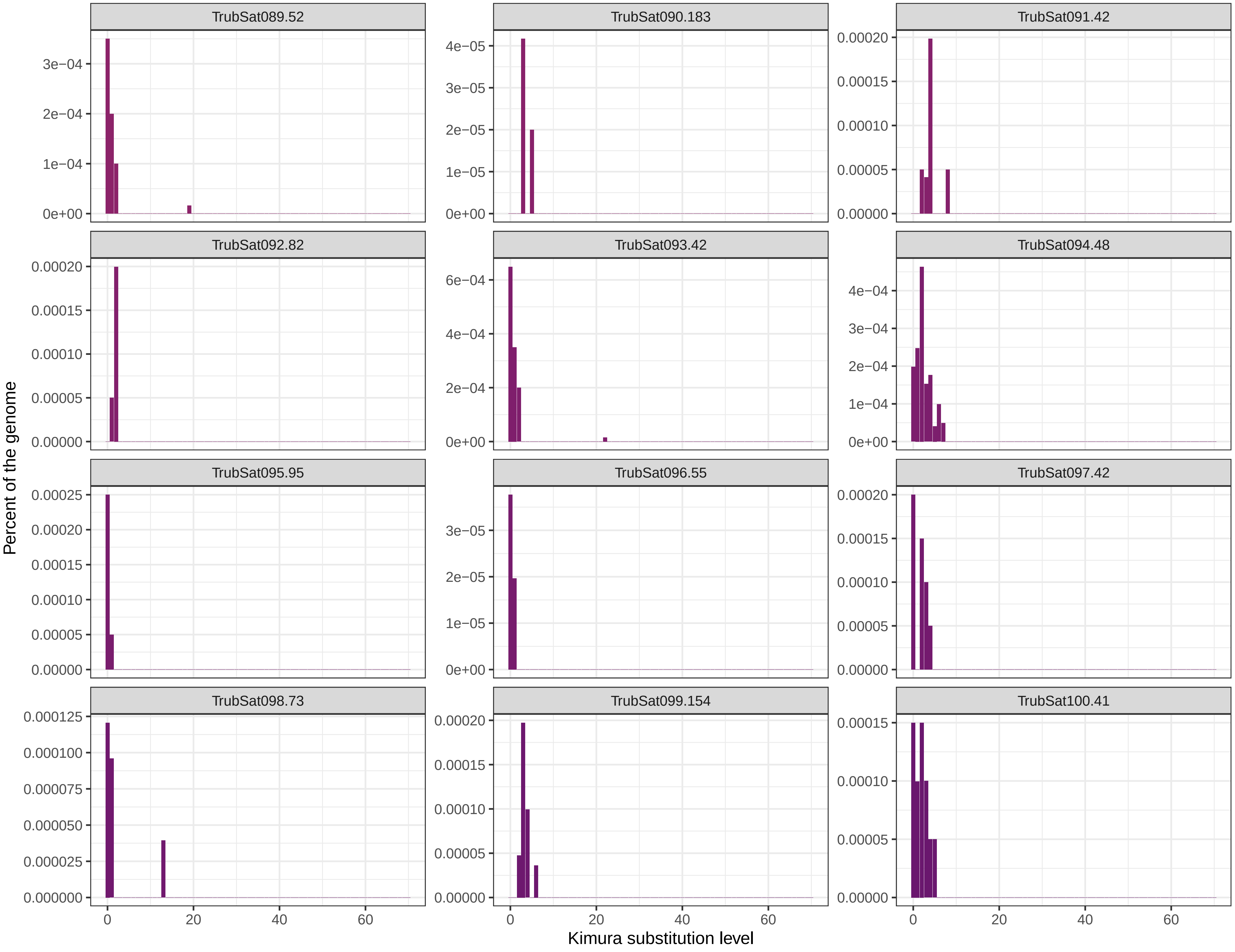
TrubSat089.52
TrubSat090.183
TrubSat091.42
TrubSat092.82
TrubSat093.42
TrubSat094.48
TrubSat095.95
TrubSat096.55
TrubSat097.42
TrubSat098.73
TrubSat099.154
TrubSat100.41
Percent of the genome
Kimura substitution level
0
20
40
60

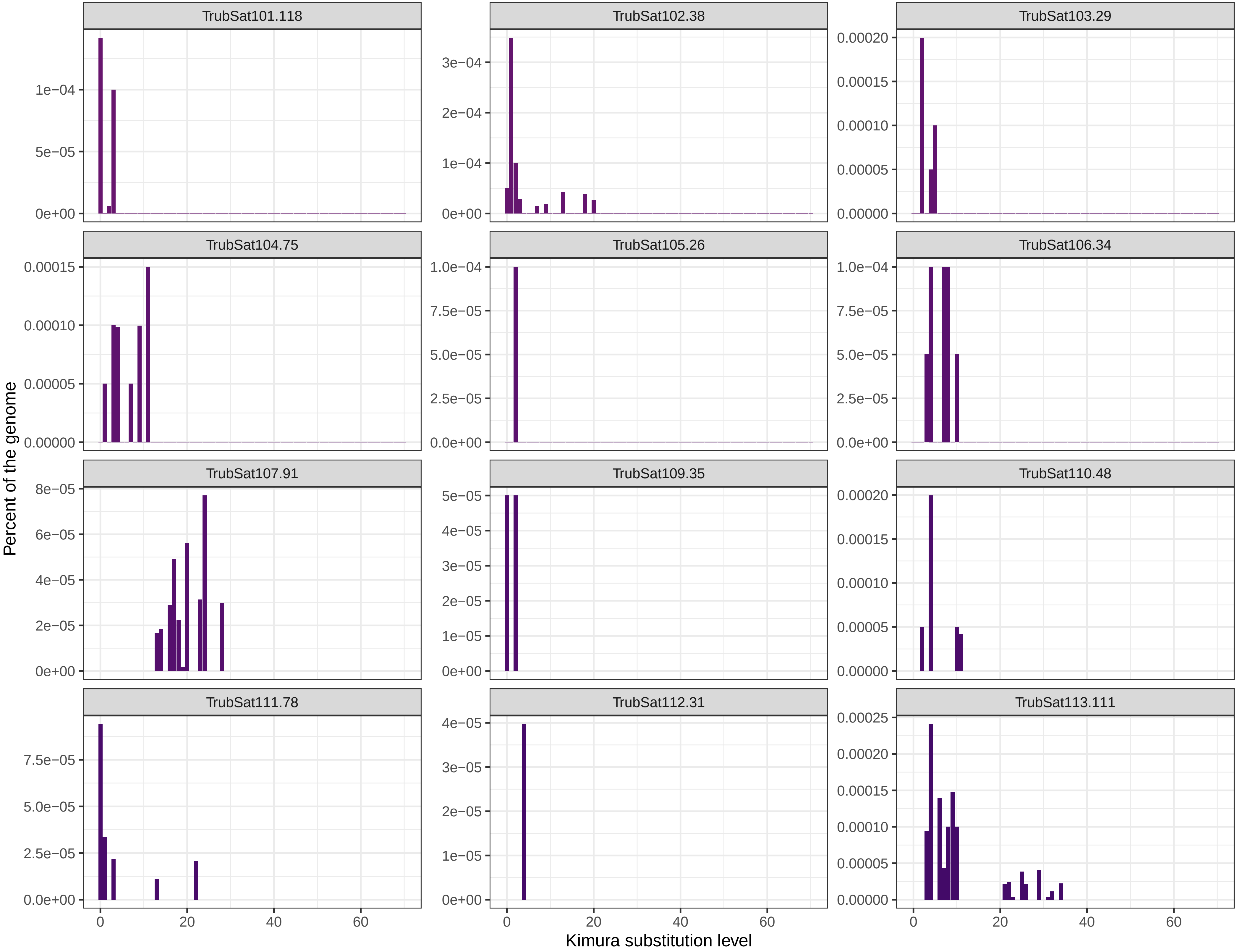
TrubSat101.118
TrubSat102.38
TrubSat103.29
TrubSat104.75
TrubSat105.26
TrubSat106.34
TrubSat107.91
TrubSat109.35
TrubSat110.48
TrubSat111.78
TrubSat112.31
TrubSat113.111
Percent of the genome
Kimura substitution level

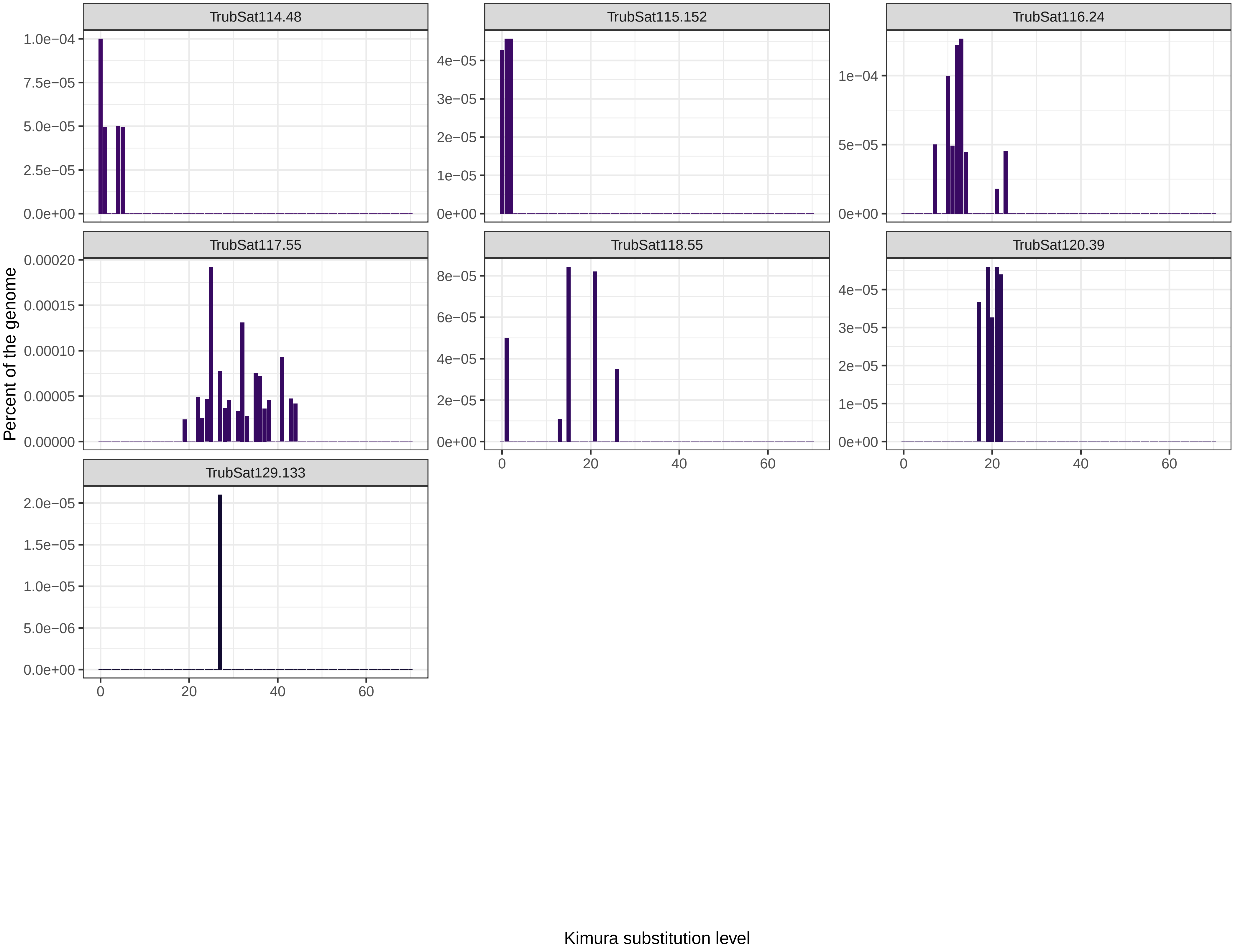
TrubSat114.48
TrubSat115.152
TrubSat116.24
TrubSat117.55
TrubSat118.55
TrubSat120.39
TrubSat129.133
Percent of the genome
Kimura substitution level
