# Supplementary material for: A new piece in the repeatome puzzle of Triatominae bugs: The analysis of Triatoma rubrofasciata reveals the role of satellite DNAs in the karyotypic evolution of distinct lineages
Source: Insect Mol Biol. 2025 Jun 27;34(6):917–28. doi: 10.1111/imb.13013 (PMC12604443; doi:10.1111/imb.13013)
Supplement: Supplementary file 4 — Figure S4. Alignments of the shared satDNAs among Triatoma rubrofasciata and others Triatomini. [file IMB-34-917-s001.pdf]

Supplementary Figure S4. Shared satDNAs

Triatoma rubrofasciata	*Triatoma infestans*	*Triatoma delpontei*	*Rhodnius prolixus*
TrubSat001-166	TinfSat075-167		
TrubSat002-9 (GATAGATTA)	TinfSat03-4	TdelSat02-8	Rpro-GATA
TrubSat003-994	TinfSat04-1000	TdelSat05-1000	
TrubSat004-97	TinfSat045-94		
TrubSat005-249	TinfSat043-242	TdelSat08-247	RproSat05-208
TrubSat009-25	TinfSat09-113	TdelSat06-25	
TrubSat015-7	TinfSat95-7	TdelSat111-7	
TrubSat018-84	TinfSat12-84	TdelSat17-84	RproSat25-84
TrubSat029-169	TinfSat70-170		

TrubSat001-166
TinfSat75-167 OQ082268

```
TrubSat001-166      GTGTGAGGAGACGACTGCTAAAATTGATGTGCTTGTAACTCCTAAAATTCTCAAGATATT     60
TinfSat75-167       GTGTGAGGAGACGACTGCTAAAATTGATGTGCTTGTAACTCCTAAAATTCTCAAGATATT     60
                    ************************************************************

TrubSat001-166      ATGCCGATATTTGGTCAAAATATACTACACTAGTATAGTATTAAACGAGCCAAAATTCAC     120
TinfSat75-167       ATGCCGATATTTGGTCAAAATATACTACACTAGTATAGCATTAAACGAGCCAAAATTCAC     120
                    ************************************** *********************

TrubSat001-166      TTAGATCTGAGCAACGGAAGTGGGTTTTTTTTGCGGGAATTTT-CAA    166
TinfSat75-167       TTAGATCTGAGCAACGGAAGTGGGTTTTTTTTGCGGGAAATTTTCAA    167
                    *************************************** *** ***

#  Percent Identity Matrix - created by Clustal2 1
#
#

     1  TrubSat001-166  100 00   97 59
     2  TinfSat75-167    97 59  100 00
```

TrubSat002-9
TinfSat03-4
TdelSat02-8
Rpro-GATA

```
TrubSat002-9 GATA - GATAGATTA -                       GATGGATTA - CATAGATTA
TinfSat03-4  GATA - GATAGATTA - GATAGTTA - GATAGGTA -
TdelSat02-8  GATA -             GATAGTTA
Rpro-GATA    GATA
```

TrubSat003-994
TinfSat04-1000 KY242403
TdelSat05-1000 OQ082083

```
TrubSat003-994      ATCTGTTGGAGCCTTTTTTGAGAAAATCTCAAAAAAAAAAAAAAAAAACATTCAGTAAAGT     60
TdelSat05-1000      ATC-GTCGGAGCCATTTTTGAGAAAATCGCAAAAAAGTAAAAAAAAACAACTAGTAAAGT     59
TinfSat04-1000      ATC-GTCGGAGCCATTTTTGAGAAAATCGCAAAAAAGTAAAAAAAAACAACTAGTAAAGT     59
                    *** ** ****** ************** *******  ***********   ********

TrubSat003-994      GGTACTTCCGGTTGAGGAATTTTGACAGAACAACTTAGTGTCGGATCCCATGGTGATACC     120
TdelSat05-1000      GGTACTTCCGGTTGAGGAATTTTGACAGAT-AACGTACTGTCAGATCCCATGGTGATACC     118
TinfSat04-1000      GGTACTTCCGGTTGAGGAATTTTGACAGAT-AACGTACTGTCAGATCCCATGGTGATACC     118
                    *****************************  *** ** **** *****************

TrubSat003-994      TAAACGGAATATCAAGTTTGAACTTTCTGCGACTTTTCGTTTTTGAGCTATGCTGTT---     180
TdelSat05-1000      TAAACGGAATATCAAGTTTCAACTTTCTACGGTTTTTCGTTTTTGAGCTATGCTGTTCAC     178
TinfSat04-1000      TAAACGGAATATCAAGTTTCAACTTTCTACGGTTTTTCGTTTTTGAGCTATGCTGTTCAC     178
                    ******************* ******** **  ************************

TrubSat003-994      ---------------------CACACACACAGACCATTTTCTAAAAACCACTTTTTTTGG     216
TdelSat05-1000      ATACATACATA----CACACACACACACACACCATTTGCTAAAAACCACTT-TTTTGG     233
TinfSat04-1000      ATACATACATACACACACACACACACACACACACACTTTGCTAAAAACCACTT-TTTTGG     237
                    *                    ********** **  *** ************* ******

TrubSat003-994      ACTCAGGGGACCTCAAAACGTATATTTCCGGTGAAAACTCGATATCGAAAATTTGACACG     276
TdelSat05-1000      ACTCAGGGGACCTCAAAACGGATATTTCCGGTGAAAACTCGATATCGAAAATTTGACACG     293
TinfSat04-1000      ACTCAGGGGACCTCAAAACGGATATTTCCGGTGAAAACTCGATATCGAAAATTTGACACG     297
                    ******************** ***************************************

TrubSat003-994      ATTACAATACTTCCTCTTACTAA-AGTAAGAGAAAGTAAAAAAAAAAAATCTGGTGTGAA     335
TdelSat05-1000      ATTACAATACTTCCTCTTACTAGGAGTAAGAGAAAGTAAAAAAAAAAAATCTGGTGTGAA     353
TinfSat04-1000      ATTACAATACTTCCTCTTACTAGGAGTAAGAGAAAGTAAAAAAAAAAAATCTGGTGTGAA     357
                    **********************  ************************************

TrubSat003-994      ACACTCACACAACTTTCTCTTACTGCAGTTTACAAAATTGTAATTGACAAATTTTATAGT     395
TdelSat05-1000      ACACTCACACAACTTTCTCTTACTCCAGTTCTCAAAATTATAATTGACAAATTTTATAGT     413
TinfSat04-1000      ACACTCACACAACTTTCTCTTACTCCAGTTCTCAAAATTATAATTGACAAATTTTATAGT     417
                    ************************ *****  ******* ********************

TrubSat003-994      TAGTATACCAAATTTAATGAAATTCGTCGGTGTTTTTATAGAGAAAATCGCGAAAACTG-     454
TdelSat05-1000      TTGTGTACCAAATTTAATGAAATTCGTC-----------------------GAAAACTGA     450
TinfSat04-1000      TTGTGTACCAAATTTAATGAAATTCGTC-----------------------GAAAACTGA     454
                    * ** ***********************                       ********

TrubSat003-994      AAAAAACTGTTCAGTAAAAACGCACTTCCGGTTTACTAATTTTCTTGAAACTCGGAATTT     514
TdelSat05-1000      AAAAAACTGTTCAGTAAAAGCGCACTTCCGGTTTACTAATTTTCTTGAAACTCGGAATTT     510
TinfSat04-1000      AAAAAACTGTTCAGTAAAAGCGCACTTCCGGTTTACTAATTTTCTTGAAACTCGGAATTT     514
                    ******************* ****************************************

TrubSat003-994      TGGCAATCTATTTATTTCTAATTAATTTTGTTTAGATGGTATAATTGTACAATCAACTA-     573
TdelSat05-1000      TGGCCATCTATTTATTTCTAATTAATTTTGTTTGGATGATAGAATTGTATAATCAACTAT     570
TinfSat04-1000      TGGCCATCTATTTATTTCTAATTAATTTTGTTTGGATGATAGAATTGTATAATCAACTAT     574
                    **** **************************** **** ** ******* *********

TrubSat003-994      ---AGCACTTTAGAGGAAAACCTAACCCCACCCCTCCCCCAAAAGTGCCT-TAAATGAAT     629
TdelSat05-1000      GATAGCACTTTAGAGAAAAACATAACCCCACCCCTCCCCTAAAAGTGCCCTTAAATGAAT     630
TinfSat04-1000      GATAGCACTTTAGAGAAAAACATAACCCCACCCCTCCCCYAAAAGTGCCCTTAAATGAAT     634
                       ************ ***** ***************** *********  *********

TrubSat003-994      TTTCCCGGAGAAATGTTTCAAATAAAAGTTGTAGCTCTTTATATGTGTAGTTGATATACA     689
TdelSat05-1000      TTTCCCAGAGAAATGTTTTAAATAAAAGTTGTAGATCTTTGTATGTGTAGTTTATATACG     690
TinfSat04-1000      TTTCCCAGAGAAATGTTTTAAATAAAAGTTGTAGATCTTTGTATGTGTAGTTTATATAGG     694
                    ****** *********** *************** ***** *********** *****

TrubSat003-994      -AGTTTAAAGAAAATCGTTGGAGCCGTTTTAGAGAAAATAGCAGAAAACTGAAAAATACT     748
TdelSat05-1000      AAGTTTCAAGAAAATCGTCGGAGCCATTTTTGAGAAAATCGCGAAAAAGTGAAAAAAATC     750
TinfSat04-1000      AAGTTTCAAGAAAATCGTCGGAGCCATTTTTGAGAAAATCGCGAAAAAGTRRAAAAAATC     754
                     ***** *********** ****** **** ******** **  **** *  **** *

TrubSat003-994      TTTCAGTAAA-----------AACGCACTTCCGGTCATCCGATTTTCTTCAAACTCGGAA     797
TdelSat05-1000      ATTTAGTAAAGTGGTAAAGTAAACGCACTTCCGGTTATCCGATTTTTTTCAAACTCGGAA     810
TinfSat04-1000      ATTTAGTAAAGTGGTAAAGTAAACGCACTTCCGGTTATCCGATTTTTTTCAAACTCGGAA     814
                     ** ******           ************** ********** *************
```

```
TrubSat003-994      TTTTCGACAACATATTTAATTCTAATTAATATTATTTAGATGATAGAATTGTGTAATAAA      857
TdelSat05-1000      T-TTCAACAACATATTTAATTCTAATTAACATTATTTAGATGACAGAATTGTCTAATAAA      869
TinfSat04-1000      T-TTCAACAACATATTTAATTCTAATTAACATTATTTAGATGAYAGAATTGTCTAATAAA      873
                    * *** *********************** ************* ******** *******

TrubSat003-994      CTGAAATAGCACTTTAGAGGAAAACCTAACCCCACCTCTCCCACAAAAGTGCCCTTAAAT      917
TdelSat05-1000      CTGAAATAGCACTTTAGATGAAAACCTAACCCCACCCCTCCCCCAAAAGTGCCCTTAAAT      929
TinfSat04-1000      CTGAAATAGCACTTTAGATGAAAACCTAACCCCACCCCTCCCCCAAAAGTGCCCTTAAAT      933
                    ****************** ***************** ***** *****************

TrubSat003-994      GAATTTTCCCGGAGAAATGTTTCAAATAAAAGTTGTAGCTCTTTGTATGTGTAGTTAATA      977
TdelSat05-1000      GAATTTTCCCGGAGAAATGTTTCAAATAAAAGTTGTAGCTCTTTGTATGTGTAGTTGATA      989
TinfSat04-1000      GAATTTTCCCRGAGAAATGTTTCAAATAAAAGTTGTAGCTCTTTGTATGTGTAGTTGATA      993
                    ********** ********************************************* ***

TrubSat003-994      TACCAAGTTTAAAGAAA    994
TdelSat05-1000      TACCAAGTTTAAAGAAA    1006
TinfSat04-1000      TACCAAGTTTAAAGAAA    1010
                    *****************


#  Percent Identity  Matrix - created by Clustal2 1
#
#

     1  TrubSat003-994  100 00   92 14   91 52
     2  TdelSat05-1000   92 14  100 00   99 20
     3  TinfSat04-1000   91 52   99 20  100 00
```

TrubSat004-97
TinfSat45-94 OQ082238

```
TrubSat004-97      CGGAAAATATCTCAAAGGATGATTTCCAGCTCTGCGGACCCAACCACAACAAAACGCCCG       60
TinfSat45-94       CGGAAAATGTCTCT---GATGCTTTCCAGCTCTGCGGAACCAACCACCACAAAACGCCCG       57
                   ******** ****    **** **************** ******** ************

TrubSat004-97      AAACCGGTCGATGACCAAAAAACTGAGCAGGCTGTCA97
TinfSat45-94       AAACCGGTCGATGACCACAAAATGAAGCAGGCTACCA94
                   ***************** ****   ********  **

#  Percent Identity  Matrix - created by Clustal2 1
#
#

     1  TrubSat004-97  100 00   88 30
     2  TinfSat45-94    88 30  100 00
```

TrubSat005-249
TinfSat43-242 OQ082237
TdelSat08-247 OQ082086

```
TrubSat005-249      GGCGCGTCAACATCTATGGTCATTGGCGCCCGGAGTCTTCTCAGTGCACTGCCGGCCTCC     60
TinfSat43-242       GGCGCGTCAACATCTATGGTCATTGGCGCCCGGAGCCTTCTCAGTGCACTGCCGGCCTCC     60
TdelSat08-247       GGCGCGTCAACATCTATGGTCATTGGCGCCCGGAGCCTTCTCAGTGCACTGCCGGCCTCC     60
                    *********************************** ************************

TrubSat005-249      GGACAGTGTTCTCTCGTTACAGACAACACTGTCCATCACCGGACAGTGAGCTGTAAGAGG     120
TinfSat43-242       GGACAGTGTTCTCTCGTTACAGACAACACTGTCCATCACCGGACAGTGAGCTGT-AGAGG     119
TdelSat08-247       GGACAGTGTTCTCTCGTTACAGACAACACTGTCCATCACCGGACAGTGAGCTGT-AGAGG     119
                    ****************************************************** *****

TrubSat005-249      CTCCTAGGAAGAGAAAATCTAAGGGCAGT--TGCTGGATTCGAACCCGGGATCTCCGCGT     178
TinfSat43-242       CTCCTAGGAAGAAAAAATCTAAGGGCAGCAC-GCTGGACTCGAACCCGGGACCTCCGCGT     178
TdelSat08-247       CTCCTAGGAAGAGAAAATCTAAGGGTAGCACAGCTGGACTCGAACCCGGGACCTCCGCGT     179
                    ************ ************ **    ****** ************ ********

TrubSat005-249      GGGAGTCCAGCACGCTAACCACTTAGCTAATCCCTTCCCTTTATTATTTTTTTTTTTTTT     238
TinfSat43-242       GGA-GTCCAG-TCGCTAACCACTGAGCTAATCCCTTCCCTTTTTTTTTTTTTTTTTT--T     234
TdelSat08-247       GGGAGTCCAGTACGCTAACCACTTAGCTATTCCCTTCCCTTTATTATTATTATTATTTTT     239
                    **  ******  *********** ***** ************ ** ** ** ** **  *

TrubSat005-249      TTTAATTTTAA   249
TinfSat43-242       --TAATTTAA-   242
TdelSat08-247       --TAATTTAA-   247
                      ****** *

#  Percent Identity  Matrix - created by Clustal2 1
#
#

     1  TrubSat005-249  100 00   95 42   95 10
     2  TinfSat43-242    95 42  100 00   95 45
     3  TdelSat08-247    95 10   95 45  100 00
```

TrubSat009-25
TinfSat09-113 KY242406
TdelSat06-25 OQ082084

```
TdelSat06-25_[5x]      AGAATGTATAACTTTGACAAATGTTAGAATGTATAACTTTGACAAATGTTAGAATGTATA   60
Trub-CL57-25_[5x]      AGAATGTAGAACTTTGGAAAAATTTAGAATGTAGAACTTTGGAAAAATTTAGAATGTAGA   60
TinfSat09-113          AGAATGTAGAACTTTGAAAAATGTTAGAATGTAGAATTTTGCGTAATATTAGAATGTATA   60
                       ******** *******  ***  ********** ** ****   **  ********** *

TdelSat06-25_[5x]      ACTTTGACAAATGTTAGAATGTATAACTTTGACAAATGTTAGAATGTATAACTTTGACAA   120
Trub-CL57-25_[5x]      ACTTTGGAAAAATTTAGAATGTAGAACTTTGGAAAAATTTAGAATGTAGAACTTTGGAAA   120
TinfSat09-113          ACTTTGGAAAACATGA------------------ATTATTAGAATGTAGAACTTTACAAA   102
                       ******  ***  * *                  *   ********** ******   **

TdelSat06-25_[5x]      ------ATGTT       125
Trub-CL57-25_[5x]      ------AATTT       125
TinfSat09-113          TACTAATTATT       113
                                **

#  Percent Identity  Matrix - created by Clustal2 1
#
#

     1  TdelSat06-25_[5x]  100 00   80 00   78 50
     2  Trub-CL57-25_[5x]   80 00  100 00   80 37
     3  TinfSat09-113       78 50   80 37  100 00
```

TrubSat015-7
TinfSat95-7 OQ082288
TdelSat111-7 OQ082187

```
>TrubSat015-7   ACAATTT
>TinfSat95-7    ACAATTT
>TdelSat111-7   ACAATTT
                *******
```

TrubSat018-84
TinfSat12-84 KY242409
TdelSat17-84 OQ082094
RproSat25-84 MW827155

```
RproSat25-84      AAACACCATATGAGAACACATACAGGCGAGAAGCCATATAAATGTACTGAATGTGATTAT 60
TinfSat12-84      AAAAGACATATGCGAACACATACAGGCGAGAAGCCATATAAATGTGGTGAATGTGATTAC 60
Trub-CL84-84      AAAAAACATATGCGAACACATACAGGCGAGAAGCCGTATAAATGTAGTGAATGCGATTAT 60
TdelSat17-84      AAAAGACATATGCGAACACATACAGGCGAGAAGCCATATAAATGTAGTGAATGCGATTAC 60
                  ***   ****** ********************** *********  ****** *****

RproSat25-84      AGTTGTACAATGTCCCAATCTTTA       84
TinfSat12-84      TGATGTACAACAACTGGAAATTTT       84
Trub-CL84-84      AGTTGTACAAAATCTGGAAATTTG       84
TdelSat17-84      AGTTGTACAACAACTGGAAATTTA       84
                   * *******   *   *  ***

#  Percent Identity  Matrix - created by Clustal2 1
#
#

     1  RproSat25-84  100.00   78.57   83.33   82.14
     2  TinfSat12-84   78.57  100.00   88.10   94.05
     3  Trub-CL84-84   83.33   88.10  100.00   92.86
     4  TdelSat17-84   82.14   94.05   92.86  100.00
```

TrubSat029-169
TinfSat70-170 OQ082263

```
TrubSat029-169      TTAAATTTACTACAATTTATGTTCTGTGGTATTTTTAGATAGGACTACTCCTTAAGGAGA      60
TinfSat70-170       TTAAATTTACTACAATTTATGTTCTGTGGTATTTTTAGATAGGACTACTCCTTAAGGAGA      60
                    ************************************************************

TrubSat029-169      TATTGCTGAAATACCAGCTAGGTGGGAGGTGAAGCTCCA-CTATTTTAGCTCATATCTTG      119
TinfSat70-170       TATTGCTGAAATACCAGTTAGGTGGAAGGTGAAGCTCCCAATATTTTAGCTCATATCTTG      120
                    ***************** ******* ************   *******************

TrubSat029-169      GTTAATATTAACTCTATGATAAAACTGTATAGAACTAAAATTGTAGAGAA 169
TinfSat70-170       GTTAATATTAACTCTATGATAAAACTGTATAGAACTAAAATTGTAGAGAA 170
                    **************************************************

#  Percent Identity  Matrix - created by Clustal2 1
#
#

     1  TrubSat029-169  100.00   97.63
     2  TinfSat70-170    97.63  100.00
```
